# Viral subversion of B-cell tolerance: mechanisms linking infection to autoimmunity

**DOI:** 10.1038/s44319-026-00959-2

**Published:** 2026-10-10

**Authors:** Michael Fraczek, Gielenny M Salem, Ivonne Melano, Xuan Ning Sharon Tan, Charlotte S M Lim, Weiqiang Chen

**Affiliations:** 1 Cleveland Clinic Lerner College of Medicine of Case Western Reserve University, Cleveland, OH USA; 2 A*STAR Infectious Diseases Labs (A*STAR ID Labs), Agency for Science, Technology and Research (A*STAR), Singapore, Singapore; 3 Lee Kong Chian School of Medicine, Nanyang Technological University, Singapore, Singapore

**Keywords:** Immunology, Microbiology, Virology & Host Pathogen Interaction

## Abstract

Viral infections are increasingly recognized as triggers of autoimmunity, yet the mechanisms linking viral infection to breakdown of B-cell tolerance remain incompletely understood. B-cell tolerance is maintained through sequential central and peripheral checkpoints that normally eliminate or silence self-reactive clones. Viral infections converge on shared mechanisms, including inflammatory cytokines, innate immune activation, molecular mimicry, and persistent antigen exposure, while also promoting polyreactivity, thereby enhancing the survival, activation, and expansion of autoreactive B cells. In this review, we propose that autoimmune outcomes are determined less by viral identity than by the immunological and anatomical context in which tolerance fails. We integrate mechanistic and clinical evidence across systemic, localized, and neurotropic viral infections to present a unifying framework for virus-induced B-cell tolerance breakdown. This framework identifies conserved principles underlying post-viral autoimmunity and provides a foundation for developing therapeutic strategies to preserve B-cell tolerance.

## Abstract (web summary)

Viral infections are increasingly recognized as triggers of autoimmunity, yet the underlying mechanisms remain incompletely understood. This review proposes that autoimmune outcomes are determined less by viral identity than by the immunological and anatomical context, and presents a unifying framework for virus-induced B-cell tolerance breakdown.

## Graphical abstract

**Figure:**
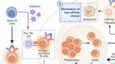

## Introduction

Autoimmune diseases arise when immune tolerance fails, allowing self-reactive lymphocytes to escape layered checkpoints that normally restrain them throughout development and activation. Among adaptive immune cells, B cells are particularly vulnerable because antibody diversification is inherently stochastic, generating an early pre-immune repertoire enriched with self-reactive specificities, many of which persist into the mature naive B-cell pool (Wardemann et al, 2003). Consequently, humoral immunity must balance rapid responsiveness with durable self-tolerance (Bonasia et al, 2021). Although genetic susceptibility influences autoimmune risk (Scepanovic et al, 2018), environmental triggers, particularly viral infections, often determine whether immune tolerance ultimately fails.

Viral infections profoundly reshape humoral immunity and can destabilize B-cell tolerance. They induce local and systemic inflammation (Castleman et al, 2022b; Gotot et al, 2012; Lee et al, 2022; Woodruff et al, 2020), provide abundant nucleic acid ligands, and frequently establish persistent or latent infections (Hoarau et al, 2010; Sivachandran et al, 2012; Tracy et al, 2012). Together, these processes expose developing and mature B cells to prolonged inflammatory and antigenic signals that promote the survival and activation of autoreactive clones (Cheemarla et al, 2021; Odumade et al, 2012; Poo et al, 2014). Diverse viral families are linked to systemic and organ-specific autoimmune diseases, including multiple sclerosis (MS) (Lanz et al, 2022; Souza et al, 2005), chronic inflammatory arthritis, autoimmune encephalitis (Cao-Lormeau et al, 2016; Liu et al, 2021), and systemic lupus erythematosus (SLE) (Chang et al, 2023; Peng et al, 2023). Yet these associations have largely been considered in isolation or interpreted as pathogen-specific phenomena, obscuring the shared immunological principles that underlie post-viral autoimmunity.

B-cell tolerance is maintained through sequential developmental and activation checkpoints that collectively eliminate, silence, or restrain autoreactive clones (Florova et al, 2024; Lang et al, 2016; Nemazee and Burki, 1989; Nossal and Pike, 1980). These checkpoints integrate antigen recognition with helper T-cell signals, innate immune sensing, and tissue-derived cytokines to regulate B-cell fates during germinal center (GC) selection and extrafollicular (EF) differentiation (Bonasia et al, 2021; Pillai and Cariappa, 2009b; Salvi et al, 2021; Zhu et al, 2025). Although highly effective, they remain vulnerable to inflammatory and antigenic perturbations introduced during viral infection. Recent studies have identified inherited defects in innate nucleic acid-sensing pathways that predispose individuals to systemic lupus and other autoimmune diseases, while also influencing susceptibility to virus-induced neuropathology (Cleaver et al, 2024; Vinuesa et al, 2023). However, these genetic models do not fully explain how wild-type viral infections disrupt B-cell tolerance in otherwise immunocompetent individuals, highlighting the need for a broader mechanistic framework.

Here, we examine how viral infections disrupt B-cell tolerance to promote autoreactivity and autoantibody-mediated disease. We first discuss the central and peripheral checkpoints that normally safeguard against autoreactivity, spanning early bone marrow development through GC and EF responses. We then examine how viruses undermine these safeguards through inflammatory cytokines, innate immune activation, molecular mimicry, polyreactivity, and persistent antigen exposure. Finally, we integrate mechanistic and clinical evidence across diverse viral systems, including SARS-CoV-2, Epstein–Barr virus (EBV), herpes simplex virus (HSV), Japanese encephalitis virus (JEV), and arthritogenic alphaviruses, into a unified framework for virus-induced B-cell tolerance breakdown. By comparing systemic, localized, and neurotropic infections, we show how the immunological and anatomical context of checkpoint failure, rather than viral identity alone, shapes the clinical manifestations of B-cell-mediated autoimmunity.

## B-cell tolerance and immune checkpoints

### Central B-cell tolerance

**Figure 1:**
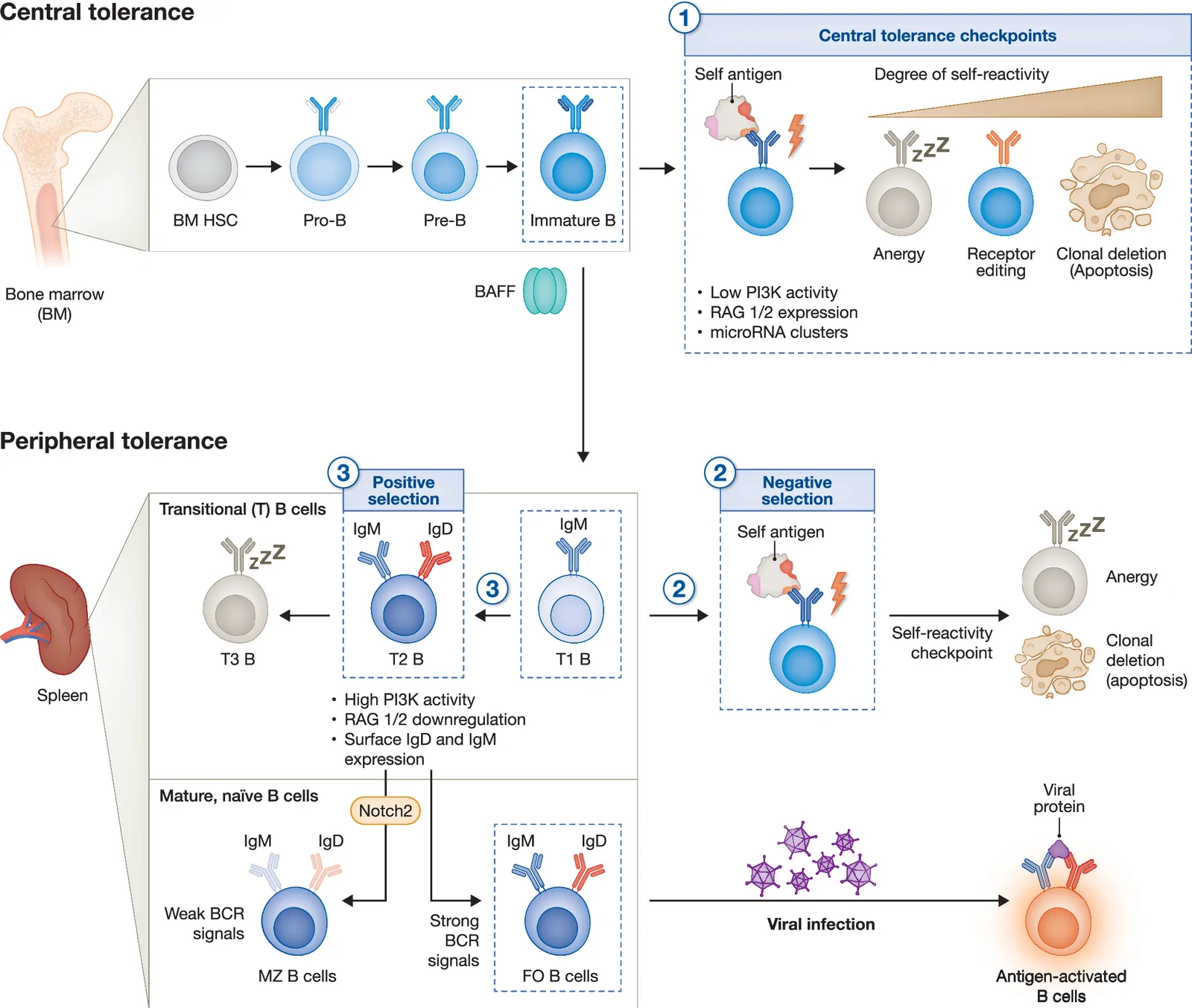
Central and peripheral checkpoints safeguarding B-cell tolerance. B-cell development begins in the bone marrow (BM), where hematopoietic progenitors differentiate sequentially into pro-B, pre-B, and immature B cells. Because most newly generated immature B cells express self-reactive B-cell receptors (BCRs), (1) central tolerance checkpoints resolve autoreactivity based on the strength of self-antigen engagement, including: (i) receptor editing; (ii) clonal deletion; or (iii) functional unresponsiveness or anergy. High-affinity interactions drive either receptor editing, mediated by RAG1/2 reactivation and secondary immunoglobulin (Ig) light-chain rearrangements to alter BCR specificity, or clonal deletion via apoptosis, whereas intermediate-affinity encounters induce functional unresponsiveness (anergy). Central tolerance is regulated by phosphoinositide 3-kinase (PI3K) signaling and microRNA networks, with transiently low PI3K activity favoring receptor editing over positive selection. Following BAFF-driven egress from the BM, immature B cells enter the circulation as transitional T1 B cells and migrate to the spleen. Here, (2) peripheral tolerance provides an additional, reinforcing layer of selection: strong autoreactive signaling induces follicular exclusion and peripheral deletion, whereas chronic low-affinity stimulation enforces anergy. Surviving cells are (3) positively selected through the T2 and T3 transitional stages, with anergic clones dynamically enriching within the T3 compartment. Ultimately, responsive T2 cells differentiate into mature naive follicular (FO) or marginal zone (MZ) B cells, which maintain divergent signaling thresholds, anatomical localizations, and peripheral tolerance constraints. Figure layout and panels are original work. Narrative schematic icons were created with BioRender.com under a standard publication license.

Central B-cell tolerance is established in the bone marrow, where newly generated immature B cells first encounter self-antigens before entering the peripheral repertoire (Cooper, 2015; Hardy and Hayakawa, 2001; Petro et al, 2002). Because 55–75% of newly generated immature B cells express self-reactive BCRs (Wardemann et al, 2003), stringent central tolerance checkpoints are required to prevent the emergence of autoreactive clones. These checkpoints represent the first encounter with self-antigens and resolve autoreactivity through three principal mechanisms: receptor editing, clonal deletion, and functional silencing through anergy (Fig. 1) (Florova et al, 2024; Lang et al, 2016; Nemazee and Burki, 1989; Nossal and Pike, 1980).

Immature B cells expressing productively rearranged BCRs with high-affinity binding to membrane-bound self-antigens are eliminated by clonal deletion, a mechanism first demonstrated in landmark transgenic mouse models (Nemazee and Burki, 1989; Osmond, 1993). When self-reactivity is strong but remains below the threshold for immediate deletion, receptor editing becomes the predominant tolerance mechanism (Halverson et al, 2004). This pathway involves the re-expression of recombination-activating genes 1 and 2 (RAG1/2), initiating secondary Ig light-chain (IgL) rearrangements that replace autoreactive BCR specificities with innocuous ones, thereby rescuing immature B cells from negative selection (Florova et al, 2024). Current evidence indicates that receptor editing constitutes the principal mechanism of central B-cell tolerance, whereas clonal deletion occurs primarily after receptor editing has been exhausted (Halverson et al, 2004). As a result of these stringent selection processes, fewer than 10–20% of developing bone marrow B cells successfully mature and exit into the peripheral circulation (Osmond, 1993).

Developing B cells that encounter soluble self-antigens with intermediate affinity escape deletion but instead become functionally silenced through clonal anergy (Nossal and Pike, 1980). This state is characterized by selective downregulation of surface IgM and attenuated intracellular responsiveness following BCR engagement (Hartley et al, 1991; Kirchenbaum et al, 2014; Nossal and Pike, 1980). Rather than being physically eliminated, anergic B cells remain functionally restrained and localize preferentially to the T–B cell border of secondary lymphoid tissues because of altered chemokine receptor expression (Fulcher et al, 1996). In the absence of cognate T-cell help, these cells are excluded from lymphoid follicles and undergo apoptosis within ~72 h (Fulcher et al, 1996). Importantly, this functional unresponsiveness is reversible rather than permanent. Classic adoptive transfer studies demonstrate that timely cognate T-cell help rescues autoreactive B cells from deletion, allowing both immature and mature B cells to enter splenic follicles, seed GCs, and participate in normal humoral responses (Cook et al, 1998; Fulcher et al, 1996).

Central B-cell tolerance is further regulated by intracellular signaling networks, particularly the phosphoinositide 3-kinase (PI3K) pathway. Elevated PI3K signaling promotes immature B-cell survival and positive selection, whereas transient PI3K inhibition permits Foxo1 activation and subsequent induction of RAG1/2 to facilitate receptor editing (Nemazee, 2017; Verkoczy et al, 2007). MicroRNAs provide an additional layer of regulation by fine-tuning this signaling threshold. The miR-19 component of the miR-17–92 cluster suppresses the phosphatase and tensin homolog (PTEN), thereby sustaining PI3K signaling, promoting positive selection, and limiting receptor editing (Lai et al, 2016). Conversely, miR-148a enhances the survival of autoreactive B cells by suppressing key pro-apoptotic regulators, including Gadd45α, PTEN, and Bim, a mechanism implicated in SLE and lupus-prone mouse models (Gonzalez-Martin et al, 2016).

Collectively, central tolerance eliminates or silences the majority of autoreactive B cells, reducing autoreactivity from ~75% in immature bone marrow B cells to around 40% upon peripheral egress (Wardemann et al, 2003). This early checkpoint shapes the pre-immune repertoire by removing pathological self-reactivity while preserving protective antiviral specificities (Florova et al, 2024). Disruption of these mechanisms permits autoreactive clones to enter the peripheral immune compartment, where they must be restrained by peripheral tolerance checkpoints to prevent systemic autoimmunity (Wardemann et al, 2003).

### B-cell peripheral tolerance

Following bone marrow egress, immature B cells enter the circulation as transitional (T1) B cells, whose survival and maturation depend critically on B-cell activating factor (BAFF) (Batten et al, 2000; Schiemann et al, 2001) (Fig. 1). BAFF receptors (BAFF-R) expression is higher on non-autoreactive than autoreactive immature B cells and correlates with surface IgM expression and tonic BCR signaling (Rowland et al, 2010). These transitional cells progress through T1 and T2 stages before differentiating into mature naive follicular (FO) and marginal zone (MZ) B cells, a process collectively referred to as positive B-cell selection (Cariappa et al, 2001; Carsetti et al, 1995; Lechner et al, 2021; Wols et al, 2010).

During the T1 stage, B cells undergo an additional round of peripheral selection based on the avidity of their BCR for peripheral self-antigens. Strong autoreactive signaling at this checkpoint triggers negative selection via clonal deletion, whereas sub-threshold or chronic, low-affinity interactions drive cells toward anergy rather than developmental progression (Carsetti et al, 1995; Loder et al, 1999). Surviving, non-autoreactive T1 cells differentiate into T2 B cells, characterized by co-expression of surface IgM and IgD, alongside a heightened responsiveness to tonic survival factors, serving as the immediate precursors to mature naive populations (Petro et al, 2002). Using a Nur77-eGFP reporter system to track endogenous BCR signaling strength, IgD expression on naive B cells has been shown to selectively dampen chronic autoreactive signaling and maintain cellular quiescence, thereby restricting aberrant differentiation into autoantibody-secreting cells (Noviski et al, 2018). A second peripheral checkpoint is represented by the T3 B-cell compartment, which is enriched for functionally anergic, self-reactive B cells. These clones exhibit reduced BCR responsiveness despite high baseline signaling activity, fail to enter the conventional mature pool, and preferentially recognize chronically present self-antigens (Liubchenko et al, 2012; Merrell et al, 2006). Collectively, these peripheral checkpoints continuously eliminate highly autoreactive clones while maintaining a restrained pool of anergic self-reactive B cells under strict signaling control.

Mature FO and MZ B cells represent functionally and anatomically distinct naive B-cell populations that operate under different tolerance constraints. FO B cells reside primarily within splenic follicles, where they participate in T cell-dependent immune responses and remain subject to stringent regulation through BCR signaling threshold and competition for survival signals. Although a subset of recirculating FO B cells can also occupy a perisinusoidal niche in the bone marrow and contribute to T-independent responses against blood-borne pathogens under specific conditions (Cariappa et al, 2005; Pillai and Cariappa, 2009a), their differentiation and activation remain tightly regulated. In contrast, innate-like MZ B cells localize to the splenic marginal zone, exhibit lower BCR signaling thresholds, and develop through a Notch2-dependent pathway that is dispensable for FO B cells (Pillai and Cariappa, 2009b). Their strategic localization enables rapid T cell-dependent and T cell-independent responses against blood-borne antigens (Cerutti et al, 2013; Weller et al, 2023; Zerra et al, 2021). Despite these functional differences, both FO and MZ B cells remain subject to peripheral tolerance mechanisms that limit activation of autoreactive clones while preserving rapid protective immunity.

Despite the collective efficiency of central tolerance checkpoints, ~40% of newly emigrated transitional B cells retain some degree of autoreactivity, which is systematically pruned down to roughly 20% within the mature peripheral pool (Goodnow et al, 1989; Wardemann et al, 2003). Autoreactive B cells that encounter cognate self-antigens in secondary lymphoid organs are censored continually through follicular exclusion; their inability to compete effectively for follicular entry and limiting survival factors leads to rapid apoptosis outside the lymphoid follicles (Wardemann et al, 2003; Yau et al, 2013). Recent evidence confirms that the periphery, rather than the bone marrow, serves as a major site of immature and transitional B-cell apoptotic clearance (Simpson et al, 2024). Residual autoreactivity that escapes deletion is silenced through clonal anergy, whose long-term maintenance requires continuous antigen engagement and sustained BCR occupancy (Gauld et al, 2005). In addition, antigen receptor revision during ongoing immune responses can further reshape peripheral BCR specificities to diminish autoantibody generation post-activation (Wang and Diamond, 2008). Collectively, peripheral tolerance functions as a dynamic checkpoint that continuously restrains autoreactive clones escaping central tolerance while preserving protective humoral immunity.

### Canonical GC responses and immune checkpoints

**Figure 2:**
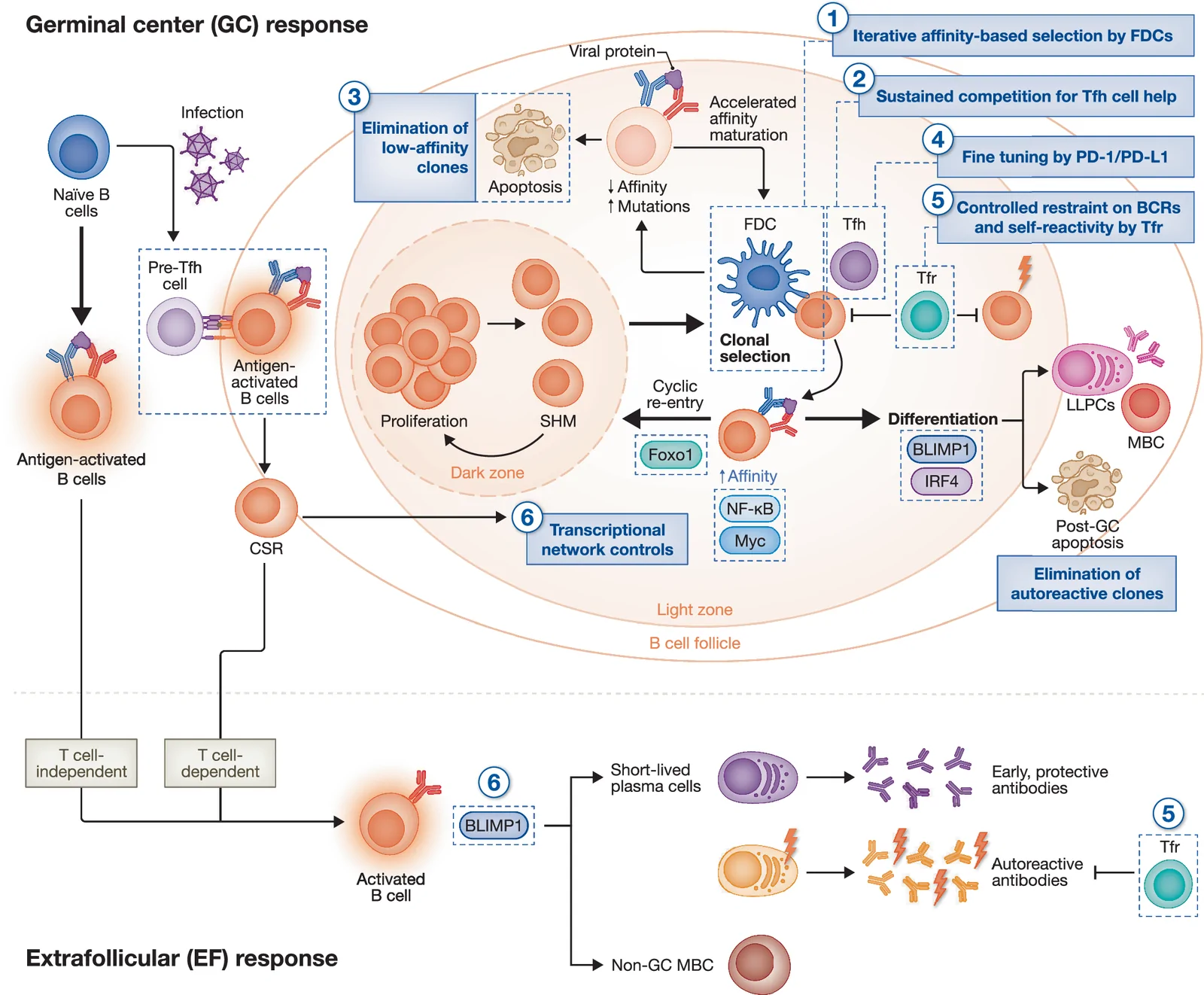
Spatiotemporal checkpoints and transcriptional dynamics of the germinal center (GC) and extrafollicular (EF) responses. Following activation and T–B priming, B cells undergo class-switch recombination (CSR) before GC commitment. Within the mature GC, B cells cycle between the dark zone (DZ), a dedicated site for rapid clonal proliferation and somatic hypermutation (SHM), and the light zone (LZ). Positive selection occurs within the LZ, where B cells undergo (1) iterative, affinity-based screening on follicular dendritic cells (FDCs) and (2) compete for limiting follicular helper T (Tfh) cell signals; (3) clones failing to secure competitive survival cues or those acquiring deleterious mutations undergo apoptosis. (4) Intra-GC selection thresholds are fine-tuned by the PD-1/PD-L1 axis and (5) regulated by follicular regulatory T (Tfr) cells. Coordination by (6) reciprocal transcriptional networks follows, wherein Foxo1 maintains the DZ program, and CD40/BCR-mediated NF-κB signaling allows Myc expression for LZ positive selection. Selected clones undergo IRF4-mediated downregulation of BCL6 to drive BLIMP1-dependent plasma cell differentiation. Deletion of somatically mutated autoreactive clones occurs at a post-GC apoptotic checkpoint to restrict entry into the memory B cell (MBC) and long-lived plasma cells (LLPCs) pools. Alternatively, early extrafollicular (EF) responses bypass the GC to rapidly produce short-lived plasma cells and non-GC MBCs. Figure layout and panels are original work. Narrative schematic icons were created with BioRender.com under a standard publication license.

The GC is the primary site of affinity maturation, generating memory B cells (MBCs) and long-lived plasma cells (LLPCs) that sustain durable humoral immunity (He and Vinuesa, 2024; Phan et al, 2006). GC B cells dynamically transit between the dark zone (DZ), where rapid proliferation and somatic hypermutation (SHM) occur, and the light zone (LZ), where FDCs and T follicular helper (Tfh) cells orchestrate clonal selection and GC maintenance (Fig. 2) (Baumjohann et al, 2013; Cyster and Allen, 2019; Hwang et al, 2015; Jacob et al, 1991; Victora and Nussenzweig, 2022; Young and Brink, 2021). While historical models placed class-switch recombination (CSR) alongside SHM in the DZ, modern tracking shows that CSR occurs predominantly prior to mature GC commitment during initial T–B cell priming and is minimized within the GC proper (Roco et al, 2019). In the LZ, productive BCR signaling is essential for GC B-cell survival and positive selection, directly coupling antigen acquisition to clonal fitness and continued GC participation (Chen et al, 2023). GC maintenance and exit are governed by transcriptional networks centered on BLIMP1, which antagonizes BCL6 and cooperates with IRF4 to promote plasma cell differentiation and follicular exit (Conter et al, 2025). Recent work using virus-like particles further challenges the classical model of rapid elimination of low-affinity clones, demonstrating that these cells can persist despite stringent competition and undergo accelerated affinity maturation (Li et al, 2025).

B-cell fate decisions during T-dependent responses are determined by the strength and integration of BCR signaling, T–B cell interactions, immune imprinting, and antigen exposure history. Depending on these inputs, selected GC B cells either re-enter the dark zone for additional rounds of selection or exit the follicle to differentiate into ASCs (Ferapontov et al, 2023; Salem et al, 2025; Staniek and Rizzi, 2025). This balance is governed by opposing transcriptional networks; Foxo1 activity enforces the DZ phenotype, whereas CD40-mediated NF-κB downregulates Foxo1 to permit Myc-driven positive selection in the light zone (Luo et al, 2018). Ultimately, the induction of IRF4 acts as a switch that overrides the GC master regulator (BCL6) to drive terminal ASC differentiation (Luo et al, 2018; Scharer et al, 2020; Ubieta et al, 2017). During initial priming, only a subset of activated B cells adopts a terminal ASC fate, whereas non-ASC populations retain accessible chromatin structures and transcriptional programs that poise them for alternative memory or GC-associated programs (Scharer et al, 2020).

Although SHM inevitably generates autoreactive BCR specificities, the accumulation of pathogenic autoantibodies is constrained by a combination of stringent affinity-based competition and discrete peripheral checkpoints. Prior to GC formation, early B-cell expansion is restricted by a pre-GC checkpoint at the T–B border, where competition for limiting T-cell help preferentially selects B cells with high-affinity antigen engagement (Schwickert et al, 2011). Within the mature GC, a positive-selection checkpoint operates through antigen acquisition from FDCs and competition for limiting Tfh cell help, preferentially expanding B cells with superior affinity for the immunizing antigen (Schwickert et al, 2011; Shulman et al, 2013). In contrast, non-selected clones or those acquiring deleterious mutations following activation-induced cytidine deaminase (AID) activity undergo apoptosis because they fail to receive sufficient competitive survival signals (Mayer et al, 2017). Crucially, because GC selection tracks antigen affinity rather than self-reactivity per se, some high-affinity autoreactive variants can evade localized deletion and participate in the GC reaction. Consequently, GC tolerance is reinforced by a post-GC apoptotic checkpoint that selectively eliminates mutated autoreactive MBCs and plasma cells (Mayer et al, 2020).

Disruption of this post-GC apoptotic control, such as enforced Bcl-2 expression, permits survival of these mutated autoreactive clones. These autoreactive clones therefore evade peripheral deletion, driving anti-nuclear antibody production, systemic autoimmunity, and increased susceptibility to lymphomagenesis (Mayer et al, 2020). Similarly, impaired downstream B-cell apoptosis leads to delayed-onset autoimmune phenotypes that are rescued by the deletion of Bruton’s tyrosine kinase (Btk), underscoring the intersection of BCR-proximal signaling thresholds and peripheral selection (Wright et al, 2021).

This multi-layered regulation is further fine-tuned by T cell-mediated mechanisms. A parallel immune checkpoint is provided by follicular regulatory T (Tfr) cells, which suppress aberrant B-cell activation to limit the expansion of autoreactive GC clones (Tulyeu et al, 2025). Immune checkpoint pathways, including PD-1/PD-L1, establish an additional checkpoint regulating Tfh-mediated positive selection within the LZ, preventing preferential survival of poorly selected or bystander B-cell clones (Garcia-Lacarte et al, 2021; Good-Jacobson et al, 2010; Shi et al, 2018). Together, these overlapping checkpoints couple affinity maturation to immune tolerance, ensuring that high-affinity protective antibodies are generated while limiting the emergence and persistence of autoreactive GC-derived clones.

### Extrafollicular (EF) responses and immune checkpoints

EF B-cell responses arise outside B-cell follicular structures and can be initiated by both T cell-dependent and T cell-independent antigens (Lam and Baumgarth, 2023; Staniek and Rizzi, 2025; Sweet et al, 2011). Although generating a less diverse repertoire than GC reactions, EF pathways can generate class-switched antibodies and early memory B-cell subsets, providing a vital first line of humoral defense during acute infection (Elsner and Shlomchik, 2025; Woodruff et al, 2020). Compared with GC responses, EF reactions are characterized by accelerated plasmablast differentiation, limited anatomical compartmentalization, and reduced dependence on follicular helper T cells, enabling early antibody production (Eisenbarth et al, 2025). During early immune responses, stronger BCR signaling preferentially directs activated B cells toward EF plasmablast differentiation, whereas weaker or more sustained signaling favors GC entry and affinity maturation (Paus et al, 2006). Thus, BCR signal strength constitutes an early fate checkpoint that partitions activated B cells between rapid EF immunity and GC-mediated affinity maturation.

During acute immune responses, EF reactions typically proceed with minimal SHM, generating antibodies that remain close to their germline configuration (Austin et al, 2019; Woodruff et al, 2020). Although this enables rapid humoral protection, it is also associated with a high frequency of autoreactive specificities that have escaped earlier tolerance checkpoints (Zhu et al, 2025). In healthy individuals and during acute infection, this autoreactivity is transient and primarily reflects the rapid activation of pre-existing self-reactive B-cell clones rather than the de novo generation of autoreactive antibodies through GC-dependent mutational diversification (Wardemann et al, 2003; Woodruff et al, 2020). In contrast, chronic autoimmune diseases such as SLE are characterized by dysregulated EF responses that express substantial AID activity, undergo extensive SHM, and support sustained clonal expansion (Elsner and Shlomchik, 2020; Jenks et al, 2019; William et al, 2002). This pathogenic transition is driven by specialized T peripheral helper (Tph) cells, which provide IL-21 and CXCL13 to promote local B-cell maturation and expansion within inflamed tissues (Bocharnikov et al, 2019; Rao et al, 2017). Current evidence suggests that the strong association between EF responses and pathogenic autoantibody production reflects the relative absence of the stringent affinity- and survival-based checkpoints that characterize GC responses, rather than active positive selection of autoreactive clones. However, direct experimental comparisons of these checkpoint stringencies remain limited.

EF responses are therefore not intrinsically pathogenic but are highly dependent on their inflammatory context. Sustained innate immune signaling, particularly through endosomal TLR7, promotes robust antiviral EF expansion following vaccination (Yin et al, 2023). During influenza infection, Myeloid differentiation primary response 88 (MyD88)/TRIF-dependent signaling and downstream NF-κB/IRF4 activation rapidly induce protective neutralizing plasmablast responses before GC formation (Lam and Baumgarth, 2023). Conversely, prolonged TLR4 activation following immunization shifts B-cell fate away from GC differentiation and toward the EF pathway, highlighting innate inflammatory cues as critical determinants of EF fate decisions (Lam and Baumgarth, 2023). Collectively, antigen persistence, pattern-recognition receptor signaling, inflammatory cytokines, and the availability and quality of extrafollicular T cell help constitute key immune checkpoints that determine whether EF responses remain protective or progress toward pathogenic autoreactivity.

A central unresolved question is which molecular checkpoints distinguish transient protective EF responses from the sustained pathogenic circuits that characterize chronic autoimmunity. Current studies of EF-like or atypical B-cell populations rely largely on phenotypic inference and disease association rather than direct lineage tracing (Woodruff et al, 2020), limiting definitive identification of their developmental origins. Defining the regulatory circuits and immune checkpoints that constrain EF B-cell activation will therefore remain essential for understanding how rapid antiviral immunity is balanced against long-term self-tolerance.

## Mechanisms of B-cell subversion by viruses

**Figure 3:**
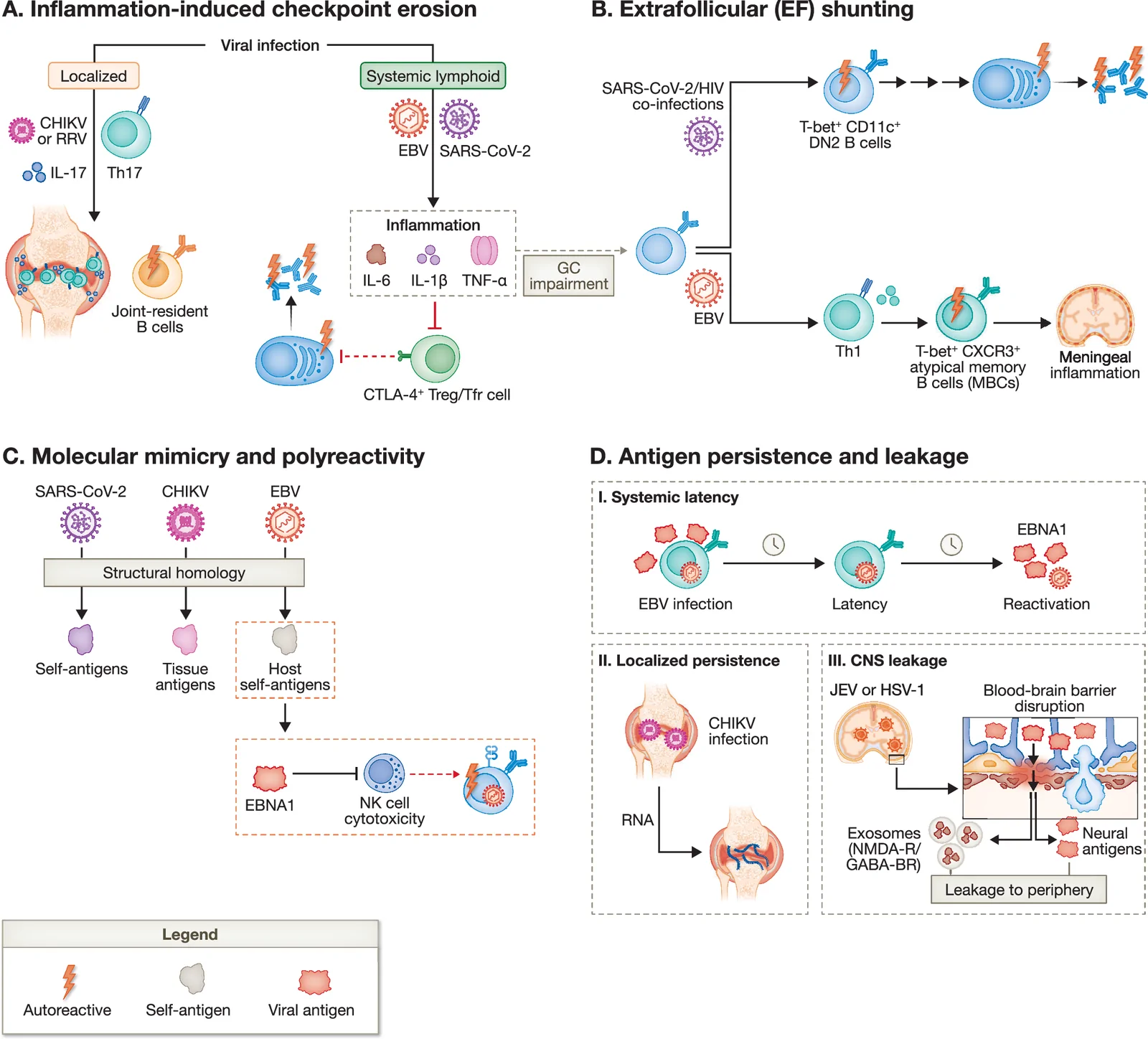
Mechanisms of viral-induced humoral autoimmunity. Viruses subvert humoral immune checkpoints and accelerate autoimmunity through four convergent operational pathways. (**A**) Inflammation-induced checkpoint erosion: Virus-induced inflammatory cytokine storms following either SARS-CoV-2 or EBV infection reprogram B-cell activation thresholds and impair regulatory networks governed by T reg and Tfr cells, which normally rely on CTLA-4-mediated mechanisms to suppress autoantibodies. Similarly, sustained Th17 cell infiltration and localized IL-17 expression following chronic CHIKV or RRV infections fuel joint-resident autoreactive B-cell expansion. (**B**) Extrafollicular (EF) shunting: Severe infections bypass conventional germinal center (GC) selection to favor the EF pathway, expanding pre-existing autoreactive B-cell pools. This was exemplified by the prominent expansion of autoreactive T-bet^+^ CD11c^+^ double-negative (DN2) B cells during critical SARS-CoV-2 infection, and the expansion of an autoreactive subset of T-bet^+^, CXCR3^+^ atypical memory B cells (MBCs) during EBV infection that drives meningeal inflammation in multiple sclerosis. (**C**) Molecular mimicry: Structural homologies between viral and self-antigens trigger cross-reactive, autoimmune cascades across multiple viruses (including SARS-CoV-2, CHIKV, and EBV), an axis further exacerbated by EBV-mediated suppression of NK cell cytotoxicity that prevents the clearance of autoantibody-producing B cells. (**D**) Antigen persistence and leakage: Chronic antigen exposure overrides peripheral checkpoints to sustain autoreactive B-cell survival via (i) systemic latency, (ii) localized persistence, and (iii) CNS leakage. (i) Latent EBV reservoirs within MBCs maintain EBNA1 mimic immunogen drive. (ii) Retention of CHIKV viral RNA in synovial tissue provides a chronic peripheral antigen source. (iii) Neurotropic JEV and HSV-1 infections cause blood-brain barrier (BBB) disruption, driving the outward egress of sequestered neural antigens into the peripheral circulation via meningeal lymphatics. Exosome-mediated release of NMDA-R and GABA-BR antigens is an additional vesicular route for autoreactive B-cell priming. Figure layout and panels are original work. Narrative schematic icons were created with BioRender.com under a standard publication license.

Although B-cell tolerance is remarkably robust, many viruses have evolved convergent strategies to bypass or erode the immune checkpoints that normally constrain autoreactive B cells, thereby reshaping B-cell differentiation and function. Viral subversion of B-cell tolerance is increasingly recognized as a determinant of acute and chronic infection outcomes and a contributor to virus-associated autoimmunity (Dougan et al, 2013; Melzi et al, 2016; Salimzadeh et al, 2018; SoRelle et al, 2022). In immune-privileged sites such as the CNS, inflammatory cues promote B-cell infiltration and expansion of autoreactive clones, amplifying tissue pathology (Jelcic et al, 2025; van Langelaar et al, 2019). Mechanistically, viruses promote EF differentiation, sustain antigen stimulation, and erode multiple immune checkpoints, collectively driving the breakdown of B-cell tolerance (Fig. 3). Despite these advances, fundamental questions remain regarding where and how viral infections initiate durable breaches in B-cell tolerance (Box 1).

Box 1 In need of answers Despite major advances linking viral infections to autoimmune disease, fundamental questions remain regarding how, when, and where antiviral immunity transitions into autoimmunity.

**1. Where does B-cell tolerance first fail during viral infection?**

Do autoreactive B cells emerge directly within infected tissues, inside draining lymph nodes (such as cervical lymph nodes receiving meningeal lymphatic drainage), or within distant peripheral lymphoid organs responding to systemic inflammation? Resolving this question will require integrated spatial transcriptomics, lineage tracing, and longitudinal analysis of B-cell responses.

**2. When are extrafollicular (EF) responses protective, and when do they become pathogenic?**

EF responses are indispensable for rapid early viral control, yet they are repeatedly linked to autoantibody production. Which molecular checkpoints determine whether these rapid, low-mutation responses remain self-limiting or instead progress toward durable autoreactivity?

**3. Is virus-induced tolerance failure transient or self-sustaining?**

In some clinical settings, autoantibody titers wane following viral clearance, whereas in others they persist long after infection resolution. What determines this trajectory, whether persistent antigen exposure, repeated inflammatory insults, or stable reprogramming of peripheral tolerance checkpoints?

**4. How does the tissue context shape the clinical landscape of distinct autoimmune diseases?**

Systemic infections, localized peripheral viral persistence, and neurotropic infections within immune-privileged sites are associated with strikingly divergent clinical outcomes. The mechanisms by which local anatomical and immunological context, site-specific stromal cells, and localized cytokine gradients uniquely guide B-cell fate decisions and autoantibody repertoires remain largely underexplored.

**5. Can tolerance be restored without undermining active antiviral immunity?**

A central translational challenge moving forward is identifying immune regulatory checkpoints that can be targeted to selectively restrain or delete autoreactive B cells while preserving protective pathogen-specific MBCs and LLPCs. Achieving this goal will require therapies that selectively restore B-cell tolerance through metabolic reprogramming or antigen-specific tolerance induction while preserving protective antiviral memory.

### Inflammatory milieu and checkpoint erosion

Viruses erode inflammatory immune checkpoints, subverting the mechanisms that normally enforce B-cell tolerance (Fig. 3A). During viremia, elevated type I interferon (IFN) and pro-inflammatory cytokines reprogram B-cell activation thresholds while impairing regulatory mechanisms, including regulatory T cell (T reg)-mediated suppression (Fallet et al, 2016; Moseman et al, 2016). In severe SARS-CoV-2 infection, high levels of CRP, IFN-I, IL-6, IL-18, and IL-1β correlate with poor clinical outcomes (Lavillegrand et al, 2021; Lucas et al, 2020). This cytokine storm, particularly IFN-I and IL-18, can promote EF responses that bypass selection in mature GCs, triggering the production of autoreactive antibodies (Enoksson et al, 2011; Soni et al, 2020).

Innate nucleic acid-sensing pathways further contribute to humoral dysregulation. Excessive TLR7 signaling is a well-established B cell-intrinsic driver of SLE (Cosgrove et al, 2023), acting in concert with IFN-γ and IL-21 to promote the differentiation of human naive B cells into autoreactive subsets (Jenks et al, 2018). Notably, TLR7 is an intracellular transmembrane receptor that senses single-stranded RNA (ssRNA) and its breakdown products, which are generated during infection by many viruses discussed here, including SARS-CoV-2, JEV, RRV, and CHIKV (Nazmi et al, 2014; Neighbours et al, 2012; Rudd et al, 2012; Salvi et al, 2021). A link between excessive TLR7 signaling and human autoimmunity has now been established through the identification of de novo and somatic *TLR7* gain-of-function (GoF) variants that promote survival of BCR-activated autoreactive B cells and cause severe early-onset lupus (Brown et al, 2022; Stremenova Spegarova et al, 2024; Zeng et al, 2025). This causal mechanism is further highlighted by multiple clinical cohorts identifying analogous TLR7 GoF mutations (David et al, 2024b; Menon et al, 2024; Mishra et al, 2024; Sethumadhavan et al, 2026). Similar autoimmune phenotypes arise from hyperactivating variants of its endosomal chaperone UNC93B1, highlighting TLR7 pathway dysregulation as a central checkpoint governing antiviral immunity and self-tolerance (Al-Azab et al, 2024; David et al, 2024a; Wolf et al, 2024).

Other pattern-recognition receptors, including TLR2, TLR8, TLR9, and RIG-I-like receptors, may similarly contribute depending on viral replication strategies, cell tropism, and host genetic context (Salvi et al, 2021; Yang et al, 2021; Zhao et al, 2022). In addition, HSV, although a DNA virus, can also induce autoimmunity through dual TLR2/TLR9 recognition pathways (Uyangaa et al, 2018), indicating that multiple nucleic acid-sensing mechanisms may converge on similar immunopathological outcomes. The overrepresentation of ssRNA viruses in virus-associated autoimmune phenomena, however, suggests that provision of abundant TLR7 ligands during infection may be a shared mechanistic feature rather than a coincidence.

Inflammation can compromise the regulatory control of humoral immunity. Under homeostatic conditions, T reg and Tfr cells actively suppress autoantibody production via CTLA-4-dependent regulations of co-stimulatory molecules on B cells and antigen-presenting cells (Gotot et al, 2012). Concurrently, co-inhibitory interactions fine-tune selection thresholds within the follicle. For instance, Treg cells can utilize direct surface contacts to suppress aberrant activation (Gotot et al, 2012), while intrinsic PD-1 signaling on Tfr cells serves as an upstream checkpoint that limits their suppressive capacity (Sage et al, 2013).

Downstream of these cellular checkpoints, anti-inflammatory cytokines, such as IL-10 and transforming growth factor-beta (TGF-β), act alongside localized metabolic filters to control global humoral scale (Çakan et al, 2025; Gotot et al, 2012; Komai et al, 2018). Crucially, active regulatory networks, primarily driven by T regs, normally restrict conventional responses by consuming and depleting IL-2 via high CD25 (IL-2 receptor) expression (Chinen et al, 2016). However, when severe viral inflammation disrupts this balance, unchecked IL-2 availability directly drives EF plasma cell differentiation and metabolic activation via the mTOR–AKT axis, accelerating the production of low-mutation, pathogenic antibodies (Faliti et al, 2024). Collectively, disruption of these regulatory networks erodes immune checkpoints that normally constrain autoreactive B-cell activation, permitting unchecked EF differentiation and autoantibody production (Gao et al, 2015; Kinnunen et al, 2013; Zhang et al, 2016). Persistent CD4^+^ T cell activation, including elevated IL-17 and Th17 infiltration, further exacerbates humoral dysregulation, as observed in chronic alphavirus-associated arthritis (Mostafavi et al, 2022). Notably, Th17 cells remain the dominant virus-specific CD4^+^ T cell subset in symptomatic patients even six years post-CHIKV infection, highlighting a potential population of interest given their capacity to interact with autoreactive B cells in other autoimmune contexts (Bai et al, 2008; Ireland et al, 2016; Liu et al, 2022).

### EF B-cell activation

Viral infection frequently skews B-cell responses towards EF activation, particularly under high inflammatory burden (Fig. 3B). In severe SARS-CoV-2 infections, GC formation is impaired, and humoral immunity is dominated by EF responses; similar patterns are observed in SARS-CoV-2 and HIV co-infection (Kaneko et al, 2020; Krause et al, 2022). Critically ill patients show early surges of IgG and IgA accompanied by expansion of T-bet^+^CD11c^+^ double-negative (DN2) B cells, a population inferred to arise through EF pathways (Castleman et al, 2022a; Cervia et al, 2021; Woodruff et al, 2020).

EF activation of B cells with autoreactive potential is also observed in other viral infections. During EBV infection, autoreactive, bystander DN2 B cells can be expanded by EBV-infected, antigen-presenting B cells from SLE patients (Younis et al, 2025), while EBV-driven T-bet^+^, CXCR3^+^ age-associated B cells (ABCs) are enriched in inflamed meninges from MS patients and promote neuroinflammation (Jelcic et al, 2025; SoRelle et al, 2025; SoRelle et al, 2022). Together, these studies suggest that viral inflammation preferentially recruits pre-existing autoreactive B-cell clones into EF differentiation pathways, thereby bypassing the stringent tolerance checkpoints normally imposed within GCs.

### Molecular mimicry and polyreactivity

Molecular mimicry bypasses antigen-specific tolerance checkpoints by allowing antiviral immune responses to cross-react with structurally related host antigens (Florova et al, 2024; Fujinami and Oldstone, 1985). Antibodies directed against viral proteins or envelope glycans can exhibit polyreactivity, cross-reacting with multiple self-antigens (Vojdani et al, 2020). Although antibodies recognizing viral envelope glycans could theoretically overcome glycan shielding in persistence-prone viruses, these glycans are often structurally indistinguishable from host glycans, making such responses inherently susceptible to autoreactivity (Florova et al, 2024). In SARS-CoV-2 infections, about 17% of IgG1 antibodies cross-react with both viral and diverse self-antigens, including nuclear, glomerular, and neuronal targets (Vojdani et al, 2020; Woodruff et al, 2022). Notably, a secreted glycoprotein of SARS-CoV-2, open reading frame 8 (ORF8) protein, functions as a viral mimic of the host IL-17A cytokine, binding the IL-17RA/RC complex to drive hyperinflammatory signaling (Wu et al, 2022).

Similarly, arthritogenic alphaviruses, such as CHIKV and RRV, share homology with joint and connective tissue antigens, including collagen α-1(II), annexin A5, α-1B-glycoprotein, complement C3, and low-density lipoprotein receptor-related protein 2 (LRP2) (Venigalla et al, 2020). Clinically, 10–20% of CHIKV-infected individuals develop chronic arthritis accompanied by the emergence of autoantibodies, including rheumatoid factor and anti-cyclic citrullinated peptide (anti-CCP) (Amaral et al, 2025; Bouquillard and Combe, 2009). Notably, disease severity correlates poorly with circulating autoantibody titers, such as rheumatoid factor IgM, anti-CCP, and anti-citrullinated α-enolase peptide 1 (CEP-1), suggesting that pathogenesis is driven by localized, tissue-resident B-cell responses within the joint microenvironment rather than systemic humoral reservoirs (Ansah-Dico et al, 2025).

EBV-driven autoimmunity further exemplifies how molecular mimicry synergizes with immune evasion (Sattarnezhad et al, 2025). The latent antigen EBNA1 shares sequence and structural similarity with CNS self-antigens involved in MS pathogenesis, including anoctamin 2, GlialCAM, and α-crystallin B (Lanz et al, 2022; Tengvall et al, 2019; Thomas et al, 2023). This molecular mimicry is complemented by additional immune evasion mechanisms. EBV-mediated suppression of NK-cell cytotoxicity prevents the clearance of activated autoreactive B cells, thereby sustaining autoantibody production (Vietzen et al, 2023). Collectively, these examples illustrate how molecular mimicry and polyreactivity cooperate with inflammation-driven EF responses to expand autoreactive B-cell pools and promote autoimmune disease.

### Antigen persistence and leakage

Persistent antigen exposure undermines peripheral tolerance checkpoints by providing continuous survival and activation signals that rescue autoreactive B cells from deletion or clonal anergy. Latent or chronic infections supply a continuous antigen drive that can rescue autoreactive B cells from localized deletion or clonal anergy. For example, EBV latency within MBCs ensures the sustained expression of the mimic immunogen EBNA1 (Sivachandran et al, 2012), while neurotropic viruses such as JEV and HSV establish persistent reservoirs within the CNS (De Chiara et al, 2019; Ravi et al, 1993).

Self-antigen leakage following infection further drives B-cell tolerance breakdown, as virus-induced lytic tissue damage and barrier disruption expose sequestered self-antigens, enabling peripheral priming of autoreactive B cells (Katz-Levy et al, 1999). Disruption of the blood-brain barrier (BBB) actively facilitates the egress of neural antigens into the periphery (Marchi et al, 2003), especially with JEV and HSV-1 infections that induce BBB permeability (Li et al, 2015; Uyar et al, 2021). Exosome-mediated release of N-methyl-D-aspartate (NMDA) receptor and GABA-B receptor-specific antigens provides an additional vesicular route for autoreactive B-cell engagement (Li et al, 2022b).

Similar processes occur in peripheral tissues, where persistent viral RNA in synovial sites following CHIKV infection provides a fluctuating antigen drive despite systemic clearance and stable antibody titers (Hoarau et al, 2010; Zhang et al, 2022). One mechanism linking CNS infection to peripheral B-cell activation is the drainage of viral antigens through meningeal lymphatics to the cervical lymph nodes, allowing peripheral presentation of CNS-derived JEV envelope proteins to naive recirculating B cells without requiring parenchymal infiltration (Li et al, 2022a). In addition, single-cell RNA sequencing (scRNA-seq) of infected CNS tissues reveals that infiltrating monocytes and macrophages constitute the dominant cell types (Ding et al, 2025; Yang et al, 2024). These findings suggest autoreactive B-cell priming is driven primarily by peripheral antigen presentation within draining secondary lymphoid organs rather than direct intraparenchymal B-cell infiltration. Collectively, persistent antigen exposure and self-antigen leakage erode peripheral tolerance checkpoints, providing a common mechanism through which viral infections promote chronic autoreactive B-cell responses and long-term autoimmune pathology.

## Case studies: viral disruption of immune tolerance

Viral infections provide a unifying framework for understanding both systemic and organ-specific autoimmunity. Across diverse pathogens, a common principle emerges: viruses act as tolerance-disrupting agents, while the breadth of systemic immune activation, precise tissue tropisms, and localized persistence profiles dictate the clinical autoimmune phenotype.

### Systemic tolerance breakdown: SARS-CoV-2

Among individuals without a prior history of autoimmunity, SARS-CoV-2 infection increases the risk of developing a new-onset autoimmune disease by about 40% (Tesch et al, 2023), affecting multiple organ systems. These include the CNS (MS), endocrine and exocrine glands (Graves’ disease and Sjögren’s syndrome), metabolic organs (type 1 diabetes, celiac disease, and pernicious anemia), connective tissues and joints (rheumatoid arthritis and ankylosing spondylitis), and the hematologic and systemic compartments (SLE and vasculitis) (Chang et al, 2023; Peng et al, 2023). In addition, multi-cohort human genetic studies confirm that host TLR7-dependent signaling serves an indispensable immunological axis for protective type I and II IFN responses during acute SARS-CoV-2 infections. Rare, deleterious, or loss-of-function *TLR7* variants on chromosome X are associated with a 5.3-fold increase in risk of developing severe COVID-19 (Butler-Laporte et al, 2022). Large-scale exome sequencing and prospective screening reveal that X-linked TLR7 deficiency strongly predisposes healthy, genetically typical males to critical COVID-19 pneumonia, particularly in patients under 60 years of age (Asano et al, 2021; Solanich et al, 2021). In patients with pre-existing immune-mediated inflammatory diseases (IMIDs), SARS-CoV-2 further amplifies chronic inflammation via viral factors such as ORF8, which functions as a pro-osteolytic agent that drives osteoclastogenesis to accelerate severe bone loss in rheumatoid arthritis (Melano et al, 2024). Taken together, these findings support a model in which failure to rapidly clear viral antigens, coupled with systemic immune activation, progressively erodes central and peripheral tolerance checkpoints to drive multisystem autoimmunity.

### Localized persistence: alphavirus-driven arthritis

Arthritogenic alphaviruses illustrate how persistent, tissue-restricted immune disruption yields localized and organ-specific autoimmunity within the joints and connective tissues (Hawman et al, 2013; Poo et al, 2014). CHIKV infection causes a chronic, rheumatoid arthritis-like disease in roughly 36% of infected individuals, with debilitating clinical symptoms persisting for months to years (Manimunda et al, 2010). Similarly, RRV induces long-lasting polyarthritis, with over 50% of exposed patients reporting chronic joint pain and stiffness three years after infection (Condon and Rouse, 1995). Persistent viral RNA and sustained tissue-resident B-cell activation provide a mechanistic basis for chronic alphavirus-associated arthritis by maintaining local antigen exposure and continually challenging peripheral tolerance checkpoints, despite the absence of widespread systemic immune dysregulation (Hoarau et al, 2010; Soden et al, 2000).

### Immune-privileged vulnerability: CNS autoimmunity

Neurotropic viruses, such as herpesviruses and orthoflaviviruses, highlight how tolerance disruption within immune-privileged tissues produces delayed, antibody-mediated disease. Specifically, EBV infection is a prerequisite for MS, conferring a 32-fold increased risk (Bjornevik et al, 2022) that remains consistent across large-scale meta-analyses (Jacobs et al, 2020). Similarly, autoimmune encephalitis can emerge during recovery from JEV infection or HSV infection. In JEV infections, up to 16% of patients develop delayed neuropsychiatric symptoms despite systemic viral clearance, often accompanied by the emergence of pathogenic autoantibodies targeting neuronal surface antigens (Liu et al, 2021; Ma et al, 2020). Further, 27% of patients with acute HSV-1 infection developed autoimmune encephalitis within 32 days, marked by psychosis, seizures, movement disorders, and the robust induction of autoantibodies against NMDA receptors (Armangue et al, 2018). Together, these examples demonstrate that although disruption of B-cell tolerance is a shared consequence of viral infection, the resulting autoimmune phenotype is determined by the anatomical site in which tolerance failure is initiated and sustained.

## Future perspectives

Despite substantial progress in understanding virus-induced autoimmunity, reconciling how distinct viral families disrupt B-cell tolerance and sustain humoral immunity dysregulation remains a key challenge for post-viral therapeutic design (Box 1). A central question is whether chronic autoantibody production reflects persistent, low-level antigen exposure, repeated inflammatory insults, or durable failure of peripheral tolerance checkpoints. For neurotropic viruses such as JEV and HSV, the precise pathways linking acute infection to delayed autoimmune encephalitis remain unclear. Longitudinal lineage tracing will be required to determine whether autoreactive B cells are primed within the CNS or in peripheral lymphoid tissues, and whether tolerance failure is transient or permanently imprinted. Resolving these mechanisms will expand the timeline for identifying early biomarkers and provide therapeutic windows for clinical intervention.

In arthritogenic alphavirus infections, including CHIKV and RRV, longitudinal analyses of autoantibody avidity, fine-specificity, isotype usage, and Fc-glycosylation profiles are required to define how humoral repertoire evolves alongside chronic joint pathology. Determining whether residual viral RNA or protein in synovial tissues sustains local B-cell activation, and mapping how B cells interact with Th17-driven inflammation, may reveal actionable targets to selectively interrupt the chronic, post-viral autoimmune arthropathies.

More broadly, extending high-throughput viral epitope-level mapping and deep longitudinal humoral profiling across viral families will be essential to establish direct causal links between infection, early checkpoint failure, and chronic autoantibody-mediated pathology. Such insights will enable targeted therapeutic strategies that restore immune tolerance while preserving protective antiviral immunity, including selective B-cell modulation, inhibition of pathogenic EF expansion, and antigen-specific tolerance induction following viral infection.

## Conclusions

Viral infections represent a unifying driver of autoimmune disease rather than a collection of pathogen-specific clinical associations. Diverse viruses converge on a limited number of mechanisms, including inflammatory checkpoint erosion, EF B-cell activation, molecular mimicry, and persistent antigen exposure, that disrupt sequential B-cell tolerance checkpoints and promote both systemic and organ-specific autoimmunity. Rather than viral identity alone, the magnitude of immune activation, tissue tropism, antigen persistence, and host immune context determine the resulting clinical phenotype. This unified framework reconciles diverse autoimmune outcomes within a common immunological model and provides a conceptual foundation for predicting post-viral autoimmune risk, identifying vulnerable tissues, and developing therapeutic strategies that restore immune tolerance while preserving protective antiviral immunity.

## Supplementary information

Peer Review File
