# Supplementary material for: Viral subversion of B-cell tolerance: mechanisms linking infection to autoimmunity
Source: EMBO Rep. 2026 Oct 10;27(21):6099–114. doi: 10.1038/s44319-026-00959-2 (PMC13743777; doi:10.1038/s44319-026-00959-2)
Supplement: Supplementary file 1 [file 44319_2026_959_MOESM1_ESM.pdf]

EMBO reports

Viral Subversion of B cell Tolerance: Mechanisms Linking Infection to Autoimmunity

Michael Fraczek, Gielenny Salem, Ivonne Melano, Xuan Ning Tan, Charlotte Lim, and Weiqiang Chen

Corresponding author(s): Weiqiang Chen (weiqiang.chen@ntu.edu.sg)

Review Timeline:

Submission Date:	12th Sep 25
Editorial Decision:	20th Oct 25
Revision Received:	10th Feb 26
Editorial Decision:	23rd Mar 26
Revision Received:	8th Jul 26
Accepted:	22nd Sep 26

Editor: Achim Breiling

Transaction Report:

(Note: With the exception of the correction of typographical or spelling errors that could be a source of ambiguity, letters and reports are not edited. Depending on transfer agreements, referee reports obtained elsewhere may or may not be included in this compilation. Referee reports are anonymous unless the Referee chooses to sign their reports.)

Dear Dr. Chen,

Thank you for the submission of your review article to our editorial offices. I have now received the full set of referee reports that is copied below. As you will see, all three referees state that your manuscript is interesting and timely. However, they have several concerns and suggestions to improve the submission that I kindly ask you to address in a revised manuscript.

Given the constructive referee comments, I would thus like to invite you to revise your manuscript with the understanding that all referee points will be addressed in the revised manuscript and in a detailed point-by-point response. Acceptance of your manuscript will depend on a positive outcome of a second round of review.

Please pay particular attention to the references, as indicated by referee #2 (point 1). Please cite primary research as much as possible, avoid citations to reviews and opinion articles, and make sure that the cited references fit and state what is mentioned in the text. Please make sure that the validity of each reference is checked by all the co-authors.

Revised manuscripts should be submitted within three months of a request for revision. Please contact me to discuss the revision (also by video chat) if you have questions or comments regarding the revision, or should you need additional time.

I further have these editorial requests:

- Please add up to 5 keywords to the manuscript and place these below the abstract.

- We have space for 2 more figures, and it would be important to have indeed 4 figures, as we encourage authors to maximize the use of visual elements, which will increase the accessibility of the piece to a non-specialist readership. Please consider adding 2 more figure and note the instructions regarding figures below. The present figures are very crowded. It might also be helpful to split these.

- We usually ask our authors to include a box called "In need of answers" that briefly outlines the major questions that are still open in a given field in the form of a few bullet points. These questions can be accompanied by a brief explanation of what would be needed to address them and may provide helpful towards setting the stage for future experimentation in the field. For an example see this recent review we published: https://www.embopress.org/doi/full/10.1038/s44319-024-00135-4

- Please also add callouts for the box to the manuscript text (Box 1).

- We now use CRediT to specify the contributions of each author in the journal submission system. CRediT replaces the author contribution section. Please use the free text box to provide more detailed descriptions and do NOT provide your final manuscript text file with an author contributions section. See also our guide to authors: https://www.embopress.org/page/journal/14693178/authorguide#authorshipguidelines

- Please also make sure the references and their callouts are formatted according to our reference format (with et al. for manuscripts with more than 10 authors): http://www.embopress.org/page/journal/14693178/authorguide#referencesformat

- Please make sure that all the funding information is also entered into the online submission system and that it is complete and similar to the one in the acknowledgement section of the manuscript text file.

I think this is a very interesting review and while I appreciate that incorporating the referees' suggestions will still require some work, I am convinced that the article is worth it and will benefit from it.

When submitting your revised manuscript, we will require a Microsoft Word file (.doc) of the revised manuscript text including detailed figure legends (at the very end), but without the figures.

Please provide the final figures as separate, high-resolution files as .pdf, .eps, .tif, or .jpg (one file per figure). Please finalize the drafts provided and make sure they accurately illustrate the key scientific concepts that you wish to show.

Please also note the following points:

- If there are certain aspects of your figure draft that are based upon assumptions or where the scientific data remains ambiguous (for example, schematically depicting a presumed direct protein-protein interaction, protein shape or subcellular localizations etc.) please add a comment so that we can work with you on an accurate depiction. Please ensure the directionality and nature of interactions is presented accurately.

- If the figure or single panels of the figure have been adapted from a published figure, please add this information to the figure legend (e.g., 'Adapted from...' or 'Based on...'). The editor will discuss if a reference and permission will be necessary

- Please only re-use figures or parts of a figure if this is essential for understanding the concept communicated. Often a reference to a previous paper will suffice. If the figure contains re-used images or elements of images, including schematics, micrographs or photos, please make sure that you have the permission/license to publish it (this also applies to your own previous work, if the journal you published in retains copyright. Certain 'creative commons' open access licenses, such as CC-BY 4.0, allow re-use without additional formal permissions). All re-used material must be explicitly cited.

- If you use an image data base for scientific iconography (e.g., BioRender), please let us know if you have a license that allows for publication in an academic journal. Often authors use misleading iconography for expedience. Please ensure the information shown is scientifically accurate. If in doubt, please discuss with the editor or provide a sketch so that our designers can create accurate iconography.

- For figures created using a software for editing vector objects like Inkscape, CorelDraw etc., please send the file as a PDF (or SVG, or EPS), PowerPoint or Keynote in which the labels and objects are still editable. For figures created using Adobe Illustrator, please send the Illustrator (.ai) file.

I look forward to seeing a revised version of your manuscript when it is ready. Please let me know if you have questions or comments regarding the revision.

Yours sincerely,

Achim Breiling
Senior Editor
EMBO Reports

Referee #1:

This is a timely review that summarizes the evidence and mechanisms by which viruses can override B cell tolerance checkpoints, resulting in defective adaptive immune responses and autoimmunity.

An important omission is failing to link viral infections with provision of TLR7 ligands - excessive TLR7 ligands cause systemic autoimmunity. All viruses mentioned in this study with the exception of HSV are RNA viruses. In fact, SARS-CoV-2, JEV, RRV, CHIKV, and ZIKV are all positive-sense single-stranded RNA viruses, with the ssRNA being a TLR7 ligand. The authors could comment whether they think this is a coincidence, or whether the evidence points to ssRNA viruses being more likely to elicit autoimmunity.

As detailed below, some definitions could be refined and, in some instances, it may be helpful to include references that more directly support the statements.

Specific points:

• Line 59 - review references? the references do not specifically refer to "acute" infection, nor deal with bystander T cell help.
• Line 68: Review references? in the two refs cited, there is no mention of anergy, and cytokine storm is only mentioned once with no causal link to autoAb production.
• Line 94: Immature B cells migrate to spleen, not to lymph nodes
• Lines 105-109: MZ can also be activated by T-dependent antigens
• Line 129 - TFR cells predominantly restrain activation of B cells reactive with self-proteins.
• Line 134 - EF responses also arise during T-dependent responses (see Toellner K-M et al., J.Exp.Med 1996 and 1998; Jacob and Kelsoe 1991 etc.
• Line 155 - there is no definitive evidence that AIRE plays a role in GC tolerance. To avoid confusion, best to remove this from this line, as it is already discussed in the section of central T cell tolerance. Regqrding line 155, other mechanisms of GC tolerance include the presence of TFR cells, and the need for selection by centrally tolerised TFH cells.
• Line 220 - Tregs suppress Ab production via multiple mechanisms, including their differentiation into Tfr cells, expression of CTLA-4 etc...
• Page 259 - perhaps describe (critically) the published mimicry between EBNA2 and GlialCAM, ANO2, CRYAB.

Referee #2:

This review article by Fraczek et al aims to review mechanisms by which viral infections might break tolerance and induce autoimmunity. The abstract promises to synthesize evidence "on virus-induced B cell dysregulation, providing a unifying framework for understanding post-infectious autoimmunity". Following a brief overview of B cell central and peripheral selection during development and a discussion on extrafollicular and germinal center responses the authors then posit that extrafollicular B cell response induction causes autoimmunity, as does "inflammation and "checkpoint erosion", molecular mimicry, antibody polyreactivity and finally antigen persistence. They conclude with the association between autoimmunity and SARS_COV2 infection, alphavirus infections as well as the association between EBV infection and individuals with MS. The text is enhanced by provision of 2 figures.

These arguments are not new but timely given the discussion on post-infection syndromes and their causes.

Unfortunately, despite the interesting topic, there are several major issues I have with this review:

1) Most importantly, the references provided throughout the review article are in many cases incorrect, incomplete or insufficient. This is a major problem, as opinions and believes expressed in this article are often not substantiated by primary research articles. Instead, some have references that are at best only partially addressing the topic, many are reviews and not primary literature articles. Some are recent articles but not key articles that discovered a particular mechanism. Often other opinion or review articles are cited, which is insufficient when trying to support statements made by the authors that often sounds as established facts, when they are only associations and possible mechanisms.

While I did not check all references, many of those I did check do not actually state what is indicated in the text. One example, Krause et al (2022) is cited as a paper that shows that EF responses (in contrast to GC responses) lack stringent tolerance checkpoints - a central argument of this review - yet the study described by Krause et al compares HIV infected and non-infected patients. Another (Dikiy & Rudensky (2023) does not report that unchecked B cell activation during inflammation destablises peripheral tolerance (by inhibiting Tregs) and enabling pathogenic antibody production. Bastard et al. discuss anti-type I IFN antibody induction not proinflammatory cytokine production causing B cell activation - these are references on just one page. There are many more examples.

2) The discussion on B cell responses is incorrect in its statement that EF responses are induced in a T-independent manner. Often, they are induced in a T-dependent manner, however, they can be induced T-independently.

3) The authors do not discuss hat the checkpoints of GC responses that do not exist in EF responses making EF responses so much more likely to generate autoantibodies - why? These cells do not undergo SHM - shouldn't they have a much lower likelihood of developing into self-reactive cells? The authors state this repeatedly as a central argument but it is never spelled out, nor justified. How are B cells in the GC checked against autoreactivity - what happens instead in the EF response?

4) EF responses are often highly successful in resolving infections despite strong presence (or likely because of) inflammatory signals - so what is different about EF responses that are successful and those that cause autoimmunity? EF responses per se are not all inducing autoreactivity. These are key important and unresolved questions - important to raise. Stating as a fact that EF responses have relaxed checkpoints - to my knowledge (and based on references provided by the authors) no papers are provided that prove this. Studies by Woodruff for example are associations of human disease outcomes and peripheral B cell measurements in which an extrafollicular origin is assumed - but never demonstrated.

5) At the end of the article, I did not see the "unifying framework" that is provided, rather a list of diseases and associations that may or may not be causal.

Minor comment:
1) Line 289....macrophages and T cells dominate as APCs with a reference by Ding et al? It seems unlikely that this study identified T cells as APCs?

Referee #3:

This comprehensive review explores the mechanisms by which viral infections subvert B cell tolerance and drive autoantibody-mediated autoimmunity, with emphasis on peripheral checkpoints, germinal center (GC) and extrafollicular (EF) responses, and the consequences of these processes across various viral families (e.g., SARS-CoV-2, EBV, alphaviruses, orthoflaviviruses, and herpesviruses). The review covers a rapidly evolving field and provides perspective not yet exhaustively reviewed in the literature. It is broadly relevant to autoimmunity, vaccinology, and infectious disease, and up to date, referencing some of the key findings from the last five years and addressing contemporary topics such as post-acute SARS-CoV-2 autoimmunity and post-viral neuroinflammation.

I have no major concerns regarding this manuscript. However, there are a few points that the authors should address:
1. The abstract mentions IGHV4-34 and biased atypical memory, but this is hardly revisited in the main text.

2. The accessibility of the astract could be improved by focusing on the key concepts and logic, with fewer examples of viruses and associated autoimmune manifestations.
3. A significant proportion of references are review articles (more than a third of all referenes, when in many cases a direct citation to the original publication would have been possible. I suggest revising th ebibliography to refer to primary research, especially for pivotal findings.
4. In Figure 1, the depiction of negative selection "arrows" may be misleading, as they point to non-autoreactive cells. It would be more accurate if they instead pointed to the autoreactive cell. Also, while CD40-CD40L interaction is relevant mainly to follicular B/T-dependent response, in the figure this may be presented as also applying to the marginal zone (MZ) response, which could confuse readers.

Specific points:
Line 134: It is not entirely accurate to state that EF responses are T-independent, since EF antibody class switching would require T cell help. Moreover, the primary focus in T-dependent GC reaction can occur in extrafollicular sites. This sentence could use more precise terminology.
Line 215: IFN-I is not defined. Please clarify what this refers to.
Line 262: It is not immediately obvious to the reader how NK cells specifically target and kill autoantibody-producing B cells. This warrants brief further clarification.

Response to Reviewers: EMBOR-2025-627441V1

Referee #1:

General comments

This is a timely review that summarizes the evidence and mechanisms by which viruses can override B cell tolerance checkpoints, resulting in defective adaptive immune responses and autoimmunity.

Response: We thank the reviewer for the positive and constructive feedback, which enabled us to significantly improve our manuscript through this revision. We have made a genuine effort to thoroughly address all the concerns raised, point-by-point, as described below:

An important omission is failing to link viral infections with provision of TLR7 ligands - excessive TLR7 ligands cause systemic autoimmunity. All viruses mentioned in this study with the exception of HSV are RNA viruses. In fact, SARS-CoV-2, JEV, RRV, CHIKV, and ZIKV are all positive-sense single-stranded RNA viruses, with the ssRNA being a TLR7 ligand. The authors could comment whether they think this is a coincidence, or whether the evidence points to ssRNA viruses being more likely to elicit autoimmunity.

Response: We thank the reviewer for this insightful comment. We agree that the enrichment of positive sense ssRNA viruses in the examples discussed is unlikely to be coincidental and may reflect a shared capacity to provide abundant TLR7 ligands. TLR7 sensing of viral ssRNA is well established, and excessive or dysregulated TLR7 signaling has been causally linked to systemic autoimmunity in mouse models and human diseases (Nazmi *et al*, 2014; Neighbours *et al*, 2012; Rudd *et al*, 2012; Salvi *et al*, 2021). Thus, ssRNA viruses such as SARS-CoV-2, JEV, RRV, and CHIKV may be particularly prone to triggering autoimmune phenomena by engaging this pathway. Thus, we note that viral nucleic acid sensing is not restricted to TLR7, and other pattern-recognition receptors (e.g. TLR2, TLR8, TLR9, RIG-I-like receptors) can also contribute depending on viral replication strategies, cell tropism, and host genetic context. For example, HSV, although a DNA virus, can induce autoimmunity through dual TLR2/TLR9-dependent pathways, demonstrating that multiple nucleic acid-sensing mechanisms may converge on similar immunopathological outcomes (Uyangaa *et al*, 2018).

We have revised the manuscript to explicitly discuss the role of TLR7 engagement by ssRNA viruses and to clarify that while current evidence supports a heightened autoimmune risk associated with ssRNA viruses, this likely reflects biased innate immune activation rather than an exclusive property of this viral class. We also note that while direct causal links remain to be established, it is likely that sustained or dysregulated TLR7 engagement during ssRNA viral infection lowers the threshold for autoreactive B cell activation and autoantibody production, particularly in genetically or immunologically susceptible hosts. We have added these concluding statements in the third paragraph of ***"Inflammation milieu and Checkpoint erosion"*** subsection.

As detailed below, some definitions could be refined and, in some instances, it may be helpful to include references that more directly support the statements.

Specific points:

• Line 59 - review references? the references do not specifically refer to "acute" infection, nor deal with bystander T cell help.
Response: We thank the reviewer for this comment. We have revised the text and replaced the original references with published articles that more directly support innate immune activation, pro-inflammatory cytokine release, and bystander T cell activation during acute viral infection (for the viruses mentioned in this review).

• Line 68: Review references? in the two refs cited, there is no mention of anergy, and cytokine storm is only mentioned once with no causal link to autoAb production.
Response: We thank the reviewer for pointing this issue. The originally cited references have been reviewed, and we agree that they did not adequately support the statements. We have, therefore, replaced the two references with two additional references that more directly address the points raised.

• Line 94: Immature B cells migrate to spleen, not to lymph nodes
Response: We thank the reviewer for noting this error. We have corrected the text to reflect that immature B cells migrate to the spleen rather than to lymph nodes.

• Lines 105-109: MZ can also be activated by T-dependent antigens
Response: We thank the reviewer for this clarification. We have revised the text to explicitly include T-dependent antigen-mediated activation of marginal zone B cells.

• Line 129 - TFR cells predominantly restrain activation of B cells reactive with self-proteins.
Response: We thank the reviewer for this important clarification. We have revised the text to specify that Tfr cells predominantly restrain B cell activation reactive with self-antigens.

• Line 134 - EF responses also arise during T-dependent responses (see Toellner K-M et al., 1996 and 1998; Jacob and Kelsoe 1991 etc.
Response: We thank the reviewer for pointing out this important point. We have revised the text to clarify that EF responses arise from both T-dependent and T-independent responses, and have expanded the references, including the suggested seminal work of Jacob and Kelsoe (1991).

• Line 155 - there is no definitive evidence that AIRE plays a role in GC tolerance. To avoid confusion, best to remove this from this line, as it is already discussed in the section of central T cell tolerance. Regqrding line 155, other mechanisms of GC tolerance include the presence of TFR cells, and the need for selection by centrally tolerised TFH cells.
Response: We thank the reviewer for this comment. We have removed the references to AIRE in GC tolerance to avoid confusion as its role is not definitively established and retained those that directly point to well-supported mechanisms. We have also revised the text to highlight the

established mechanisms of GC tolerance and expanded to include the presence of Tfr cell and the requirement for selection by centrally tolerized Tfh cells.

• Line 220 - Tregs suppress Ab production via multiple mechanisms, including their differentiation into Tfr cells, expression of CTLA-4 etc...

Response: We thank the reviewer for this suggestion. We have revised the manuscript to more clearly describe the multiple mechanisms by which Tregs regulate autoreactive B cells. These include PD-1-dependent inhibition and induction of apoptosis, differentiation into Tfr cells, secretion of anti-inflammatory cytokines, and engagement of inhibitory receptors. We have also emphasized that inflammatory conditions can impair T reg function, permitting unchecked humoral autoreactivity. These changes were reflected in the revised manuscript in the fourth paragraph of ***"inflammatory milieu and checkpoint erosion"*** subsection.

• Page 259 - perhaps describe (critically) the published mimicry between EBNA2 and GlialCAM, ANO2, CRYAB.

Response: We thank the reviewer for this suggestion. We have expanded the text in lines 259 to discuss molecular mimicry between EBV antigens (EBNA1) and CNS self-antigen, highlighting that EBV peptides are recognized by CSF or plasma antibodies that cross-react with these proteins. To our knowledge, there are no any peer-reviewed, published publications detailing molecular mimicry between EBNA2 and these self-antigens.

Referee #2:

This review article by Fraczek et al aims to review mechanisms by which viral infections might break tolerance and induce autoimmunity. The abstract promises to synthesize evidence "on virus-induced B cell dysregulation, providing a unifying framework for understanding post-infectious autoimmunity". Following a brief overview of B cell central and peripheral selection during development and a discussion on extrafollicular and germinal center responses the authors then posit that extrafollicular B cell response induction causes autoimmunity, as does "inflammation and "checkpoint erosion", molecular mimicry, antibody polyreactivity and finally antigen persistence. They conclude with the association between autoimmunity and SARS_COV2 infection, alphavirus infections as well as the association between EBV infection and individuals with MS. The text is enhanced by provision of 2 figures.

These arguments are not new but timely given the discussion on post-infection syndromes and their causes.

Response: We thank the review for their thoughtful evaluation and for recognizing the timeliness of our review on virus-induced B cell dysregulation. While the reviewer notes that many mechanisms we discuss have been described previously, we have revised the manuscript to emphasize novel insights, critically synthesize the literature and provide a more integrated framework for understanding post-infectious autoimmunity.

To improve the manuscript novelty, we highlighted recent advances in understanding extrafollicular B cell responses interact with checkpoint erosion and inflammatory cues by bringing

in updated references from research articles, as earlier mentioned in the revised ***"Introduction"***. We also revised relevant subsections in section, ***"B cell tolerance and Immune checkpoints"*** to provide a clearer description of central and peripheral B cell tolerance and how viruses may selectively breach these checkpoints. We also expanded and critically evaluated virus-specific molecular mimicry and its functional consequences in human diseases (***"molecular mimicry and polyreactivity"***). To improve coherence, we strengthened the integration of **SARS-CoV-2, alphaviruses, and Epstein–Barr virus**, highlighting both shared and virus-specific features. Finally, the original Figure 1 was divided into two figures (new Figures 1 and 2) to more clearly illustrate B cell tolerance and immune checkpoint concepts.

Overall, the revised manuscript no provides a more critical, mechanistic, and cohesive synthesis of virus-induced B cell dysregulation, addressing concerns on novelty, clarity, and depth. We believe that these changes improve both the conceptual and narrative flow, enhancing the utility of this review for the field.

Unfortunately, despite the interesting topic, there are several major issues I have with this review:

1) Most importantly, the references provided throughout the review article are in many cases incorrect, incomplete or insufficient. This is a major problem, as opinions and believes expressed in this article are often not substantiated by primary research articles. Instead, some have references that are at best only partially addressing the topic, many are reviews and not primary literature articles. Some are recent articles but not key articles that discovered a particular mechanism. Often other opinion or review articles are cited, which is insufficient when trying to support statements made by the authors that often sounds as established facts, when they are only associations and possible mechanisms.

While I did not check all references, many of those I did check do not actually state what is indicated in the text. One example, Krause et al (2022) is cited as a paper that shows that EF responses (in contrast to GC responses) lack stringent tolerance checkpoints - a central argument of this review - yet the study described by Krause et al compares HIV infected and non-infected patients. Another (Dikiy & Rudensky (2023) does not report that unchecked B cell activation during inflammation destablises peripheral tolerance (by inhibiting Tregs) and enabling pathogenic antibody production. Bastard et al. discuss anti-type I IFN antibody induction not proinflammatory cytokine production causing B cell activation - these are references on just one page. There are many more examples.

Response: We thank the reviewer for this careful evaluation and for highlighting concerns regarding the accuracy and appropriateness of the references. We agree that robust citation of primary research is critical to substantiate the review. In response, we thoroughly revised the manuscript by replacing references that were inappropriate, incomplete, or secondary (nearly all review and opinion articles) with primary research articles that supported their associated claims. Mis-citations have already been corrected, and additional key studies were included to substantiate mechanisms of EF and GC responses, checkpoint erosion, Treg function, and autoantibody production. For example, Krause et al (2022) was removed, as it did not support the claim of EF responses lack stringent tolerance checkpoints. The sentence referencing Dikiy and

Rudensky (2023) was revised to be more general and is now supported by primary studies (Gao et al. 2015, Kinnunen et al., 2013, and Zhang et al., 2016). Similarly, Bastard et al. (2020) and two review articles were replaced with three primary research articles that directly supported cytokine-driven B cell activation.

2) The discussion on B cell responses is incorrect in its statement that EF responses are induced in a T-independent manner. Often, they are induced in a T-dependent manner, however, they can be induced T-independently.
Response: We thank the reviewer for pointing out this important point. We have revised the text to clarify that EF responses arise from both T-dependent and T-independent responses, and have expanded the references, including the suggested seminal work of Jacob and Kelsoe (1991).

3) The authors do not discuss that the checkpoints of GC responses that do not exist in EF responses making EF responses so much more likely to generate autoantibodies - why? These cells do not undergo SHM - shouldn't they have a much lower likelihood of developing into self-reactive cells? The authors state this repeatedly as a central argument but it is never spelled out, nor justified. How are B cells in the GC checked against autoreactivity - what happens instead in the EF response?
Response: We thank the reviewer for this critical feedback and agree that the distinction between GC and EF responses with respect to autoreactivity required clearer explanation. We have significantly revised the manuscript to explicitly state that increased autoantibody production associated with EF responses does not arise from *de novo* autoreactivity generated by somatic hypermutation. Instead, GC reactions incorporate multiple tolerance checkpoints, including stringent competition for T follicular helper cell help, affinity-based selection on follicular dendritic cells, and apoptosis during iterative selection cycles, that actively eliminate or silence self-reactive B cell clones generated by SHM or entering the GC with pre-existing autoreactivity.

In contrast, EF responses typically proceed with minimal or absent SHM and therefore do not generate new autoreactive specificities at high frequency. Autoreactive EF antibodies are instead thought to derive from activation of pre-existing self-reactive B cell clones that have escaped earlier tolerance checkpoints, in the absence of several GC-specific selection mechanisms. More importantly, we have re-written the section, "***B cell tolerance and immune checkpoints***" and introduced two separate subsections on "***B cell central tolerance***" and "***B cell peripheral tolerance***". Further, two subsections were also rewritten "***Canonical Germinal Centers and its checkpoints***" and "***Extrafollicular responses and its checkpoints***" to clearly discuss GC and EF responses and to delineate the checkpoints required in both. We have significantly revised the manuscript to explicitly articulate this distinction and to clarify that our central argument is not that EF responses generate autoreactivity through mutation, but that they lack the GC-specific selection checkpoints that normally suppress autoreactive B cells. We believe these revisions directly address the reviewer's concern.

4) EF responses are often highly successful in resolving infections despite strong presence (or likely because of) inflammatory signals - so what is different about EF responses that are successful and those that cause autoimmunity? EF responses per se are not all inducing

autoreactivity. These are key important and unresolved questions - important to raise. Stating as a fact that EF responses have relaxed checkpoints - to my knowledge (and based on references provided by the authors) no papers are provided that prove this. Studies by Woodruff for example are associations of human disease outcomes and peripheral B cell measurements in which an extrafollicular origin is assumed - but never demonstrated.

Response: We thank the reviewer for this insightful and important comment. We fully agree that extrafollicular (EF) responses are not intrinsically autoreactive and can be highly effective at resolving infections, often in the context of strong inflammatory signaling. We also agree that current evidence, particularly from human studies such as Woodruff et al., is largely associative and based on phenotypic inference rather than direct lineage tracing or mechanistic demonstration of EF-specific tolerance defects.

To address this concern, we have substantially revised the EF section to avoid presenting relaxed immune checkpoints as established fact. We now explicitly frame EF tolerance as context dependent and incompletely defined, emphasize that autoreactive EF responses likely reflect activation of pre-existing self-reactive clones rather than *de novo* diversification, and highlight the distinction between protective and pathogenic EF responses as a central unresolved question. We also acknowledge the limitations of current human studies in definitively establishing extrafollicular origin or checkpoint failure. Notably, we have added Box 1 section, which contains all unresolved or outstanding questions in the field of B cell immunobiology.

We believe these revisions improve conceptual accuracy and better reflect the current state of the field.

5) At the end of the article, I did not see the "unifying framework" that is provided, rather a list of diseases and associations that may or may not be causal.
Response: We appreciate the reviewer for this comment. We have revised the final section ("***Case studies: Viral disruption of immune tolerance***") to explicitly articulate a unifying framework rather than a list of associations. The revised manuscript now frames viral infections as tolerance-disrupting events, with autoimmune phenotype determined by the scale of immune activation, viral tissue tropism or persistence, and host immune context. Each case study subsection was also rewritten and was also explicitly mapped onto this model presented in the second section, "***Mechanism of B cell subversion of viruses***". We also prepared introductory and concluding paragraphs in this subsection to clarify the causal logic underlying the associations discussed.

Minor comment
1) Line 289....macrophages and T cells dominate as APCs with a reference by Ding et al? It seems unlikely that this study identified T cells as APCs?
Response: We thank the reviewer pointing out this inaccuracy. We reviewed the original reference and corrected the text to accurately reflect the findings of both studies, which identify monocytes and macrophages as the predominant antigen-presenting cell populations.

Referee #3
This comprehensive review explores the mechanisms by which viral infections subvert B cell tolerance and drive autoantibody-mediated autoimmunity, with emphasis on peripheral checkpoints, germinal center (GC) and extrafollicular (EF) responses, and the consequences of these processes across various viral families (e.g., SARS-CoV-2, EBV, alphaviruses, orthoflaviviruses, and herpesviruses). The review covers a rapidly evolving field and provides perspective not yet exhaustively reviewed in the literature. It is broadly relevant to autoimmunity, vaccinology, and infectious disease, and up to date, referencing some of the key findings from the last five years and addressing contemporary topics such as post-acute SARS-CoV-2 autoimmunity and post-viral neuroinflammation. I have no major concerns regarding this manuscript. However, there are a few points that the authors should address:
Response: We thank the reviewer for their positive assessment if the manuscript and fo these constructive suggestions.

1. The abstract mentions IGHV4-34 and biased atypical memory, but this is hardly revisited in the main text.
Response: We agree with this point. To maintain consistency between the Abstract and the revised body of the manuscript, we have significantly revised the Abstract to remove specific references to IGH4-34, atypical MBCs, and other virus-specific details. The revised abstract focuses on the unifying framework of B cell tolerance and how multiple checkpoints have been eroded by viral infections.

2. The accessibility of the abstract could be improved by focusing on the key concepts and logic, with fewer examples of viruses and associated autoimmune manifestations.
Response: We agree with the reviewer and substantially revised the abstract to emphasize the core conceptual framework rather than enumerating specific viral examples or disease associations. We believe that the revised abstract now foregrounds conserved mechanisms of B cell tolerance disruption, improved the narrative flow of the Abstract and is a more accurate representation of the revised manuscript.

3. A significant proportion of references are review articles (more than a third of all references, when in many cases a direct citation to the original publication would have been possible. I suggest revising the bibliography to refer to primary research, especially for pivotal findings.
Response: We thank the reviewer for this careful evaluation and for highlighting concerns regarding the accuracy and appropriateness of the references. We agree that robust citation of primary research is critical to substantiate the review. In response, we thoroughly revised the manuscript by replacing references that were inappropriate, incomplete, or secondary (nearly all review and opinion articles) with primary research articles that supported their associated claims. Mis-citations have already been corrected, and additional key studies were included to substantiate mechanisms of EF and GC responses, checkpoint erosion, Treg function, and autoantibody production.

4. In Figure 1, the depiction of negative selection "arrows" may be misleading, as they point to non-autoreactive cells. It would be more accurate if they instead pointed to the autoreactive cell. Also, while CD40-CD40L interaction is relevant mainly to follicular B/T-dependent response, in the figure this may be presented as also applying to the marginal zone (MZ) response, which could confuse readers.
Response: We thank the reviewer for pointing this out. Figure 1 was redrawn to more accurately capture the central and peripheral B cell tolerance and immune checkpoints in the bone marrow and peripheral lymphoid tissues. Specifically, the original Figure 1 was divided into two figures (new Figures 1 and 2) to more clearly illustrate B cell tolerance and immune checkpoint concepts. We hope that the revised figures convey more clearly the intended tolerance and checkpoint mechanisms without ambiguity.

Specific points

Line 134: It is not entirely accurate to state that EF responses are T-independent, since EF antibody class switching would require T cell help. Moreover, the primary focus in T-dependent GC reaction can occur in extrafollicular sites. This sentence could use more precise terminology.
Response: We thank the reviewer for pointing out this important point. We have revised the text to clarify that EF responses arise from both T-dependent and T-independent responses, and have expanded the references, including the seminal work of Jacob and Kelsoe (1991) among others.

Line 215: IFN-I is not defined. Please clarify what this refers to.
Response: Thank you for pointing this out. The IFN-I (type 1 interferon) was spelled out at first mention in the manuscript.

Line 262: It is not immediately obvious to the reader how NK cells specifically target and kill autoantibody-producing B cells. This warrants brief further clarification.
Response: We appreciate the reviewer for pointing out this confusion. The experimental set-up (*ex vivo* co-culture) referenced in the research article (Vietzen et al., 2023) was added to the text to provide further clarification.

Dear Dr. Chen,

Thank you for the submission of your revised review article to our editorial offices. I have now received the full set of reports from the referees that were asked to re-evaluate the manuscript. As you will see, all three referees now support publication of the study. However, referees #1 and #3 have remaining concerns and comments that need to be addressed in a further revised manuscript.

Please pay particular attention to the references, as indicated by referee #3 ("The authors should go through the references once more to ensure their claims are based on experimental data, the references are accurate and clearly indicate where the authors suggest their own model without direct experimental support "). Again: Please make sure that the validity of each reference is checked by all the co-authors.

Please also update your contact information.

As I wrote previously, I think this is a very interesting review and while I appreciate that incorporating the referees' suggestions will still require some work, I am convinced that the article is worth it and will benefit from it.

As before, when submitting your revised manuscript, we will require a Microsoft Word file (.doc) of the revised manuscript text including detailed figure legends (at the very end), but without the figures. Please provide the final figures as separate, high-resolution files as .pdf, .eps, .tif, or .jpg (one file per figure). Please finalize the drafts provided and make sure they accurately illustrate the key scientific concepts that you wish to show.

I look forward to seeing a revised version of your manuscript when it is ready. Please let me know if you have questions or comments regarding the revision.

Best,

Achim Breiling
Senior Editor
EMBO Reports

Referee #1:

The authors have addressed most comments, but there are some areas that need revising for accuracy.

Comments on B cell anergy: lines 108. Self reactive B cells activated through the BCR but failing to receive T cell help do not persist. Very much like foreign antigen activated B cells, they migrate to the T:B borders of secondary lymphoid tissues and if they fail to receive T cell help they die within 72 hours. This needs to be clarified. Tony Basten and Barbara Fazekas de St Groth demonstrated that if self-reactive antigen-activated (so called anergic) B cells receive T cell help, they survive and mount normal responses (PMID: 9710232; PMID: 8642340), indicating that functional inactivation is effectively lack of T cell help.

Line 331 - Comments re "direct causality between sustained TLR7 signaling and autoimmunity remain to be established". This was established a few years ago with the demonstration that TLR7 GoF variants cause human lupus - Brown, G. J. et al. doi:10.1038/s41586-022-04642-z followed by multiple similar reports of TLR7 GoF variants or its chaperone UNC93B1 causing human systemic autoimmune disease:

• Menon, S. et al. Refractory Autoimmune Thrombocytopenia in an Infant with a De Novo TLR7 Gain-of-Function Variant. J Clin Immunol 45, 27, doi:10.1007/s10875-024-01824-4 (2024).
• David, C. et al. Gain-of-function human UNC93B1 variants cause systemic lupus erythematosus and chilblain lupus. J Exp Med 221, doi:10.1084/jem.20232066 (2024).
• David, C. et al. Interface Gain-of-Function Mutations in TLR7 Cause Systemic and Neuro-inflammatory Disease. J Clin Immunol 44, 60, doi:10.1007/s10875-024-01660-6 (2024).
• Mishra, H. et al. Disrupted degradative sorting of TLR7 is associated with human lupus. Sci Immunol 9, eadi9575, doi:10.1126/sciimmunol.adi9575 (2024).
• Al-Azab, M. et al. Genetic variants in UNC93B1 predispose to childhood-onset systemic lupus erythematosus. Nat Immunol 25, 969-980, doi:10.1038/s41590-024-01846-5 (2024).
• Han, X. et al. A novel *UNC93B1* gain-of-function mutation leads to TLR7 and TLR8 hyperactivation and systemic lupus erythematosus. medRxiv, 2024.2009.2011.24313360, doi:10.1101/2024.09.11.24313360 (2024).
• Stremenova Spegarova, J. et al. A de novo TLR7 gain-of-function mutation causing severe monogenic lupus in an infant. J Clin Invest 134, doi:10.1172/JCI179193 (2024).

• Tusseau, M. et al. Novel TLR7 gain-of-function variant and review of the associated disease spectrum. Journal of Human Immunity 2, doi:10.70962/jhi.20250199 (2026).
• Wolf, C. et al. UNC93B1 variants underlie TLR7-dependent autoimmunity. Sci Immunol 9, eadi9769, doi:10.1126/sciimmunol.adi9769 (2024).
• Zeng, Y. et al. Somatic gain-of-function mutation in TLR7 causes early-onset systemic lupus erythematosus. Ann Rheum Dis 84, 442-450, doi:10.1016/j.ard.2025.01.011 (2025).
• Parvaneh, N. et al. TLR7 F507S gain-of-function mutation presenting with early-onset SLE and hypertriglyceridemia: Response to JAK inhibition. Journal of Human Immunity 2, doi:10.70962/jhi.20250196 (2026).
• Sethumadhavan, A. et al. Identification of a novel TLR7 gain-of-function variant that underlies systemic lupus erythematosus. Journal of Human Immunity 2, doi:10.70962/jhi.20250194 (2026).

This type of evidence should also be cited in line 317.

Line 428. Might be worth mentioning that TLR7 is required for the response to SARS-Cov2 (several papers showing male patients with LoF genetic variants in TLR7 develop more severe disease) - Asano et al., Sci. Immunol.6, eabl4348 (2021); Solanich et al., Front. Immunol.12, 719115 (2021); Butler-Laporte et al., PLOS Genet.18, e1010367 (2022).

Referee #2:

The revised review is much improved in content and style. Specifically, primary literature now supports their conclusions and statements.

Referee #3:

The authors present a review discusses mechanisms and pathways on how viral infection contribute to B-cell mediated autoimmunity. The manuscript reviews general central and peripheral B cell tolerance including EF and GC tolerance checkpoints. The authors aim to make a unifying point on how make viruses can subvert B cell tolerance. While the review is timely and unifies some of the findings in the field, there are a few reviews on published on the topic recently, with sliglty different emphasis, such as Vinuesa, Grenov Kassiostis 2023 Science (DOI: 10.1126/science.adg6427), Cleaveer et al. Brain 2024 (DOI: 10.1093/brain/awad419).

While the manuscript has its merits, it suffers from several factual inaccuracies, poorly supported conclusions and inappropriate references to articles.

A couple of examples:

Lines 159-160
Pillai & Cariappa 2009 do not suggest or demonstrate T-independent responses taking place in bone marrow. This is a blatant error and should be corrected.

Lines 187-188
"...the dark zone (DZ), where rapid proliferation, somatic hypermutation (SHM), and class-switch recombination occur, and..." and the Figure 2
The CSR switching would require T cell help. DZ is largely devoid of T cells. The idea that GC are the major site of CSR has been challenged by a finding that large proporsion of CSR occurs prior to GC (Roco JA, et al. Class-Switch Recombination Occurs Infrequently in Germinal Centers. Immunity. 2019 Aug 20;51(2):337-350.e7. doi: 10.1016/j.immuni.2019.07.001)

Lines 209-220:
Inaccurate references throughout:
Mayer et al 2020 do not seem to demonstrate that germinal centers generate self reactive antibodies, but rather refer to earlier findings on this. In fact the message of the article seems to be rather that there is a checkpoint after GC reaction. Schwickert et al. 2011 do not investigate GC Tfh cells, but rather cues before establishing GCs. Merkenschlager et al 2021 do not seems to provide evidence for preferential selection for foreign antigen over autoantigens. Phan et al 2006 do not make distiction between autoantigens and foreign antigens.
As a result, the claims of GC-intrinsic selecton against autoreactivity e.g. "GC reactions incorporate multiple, well-defined immune checkpoints that actively constrain self-reactivity" or "Together, these checkpoints ensure that SHM-induced reactivity is efficiently eliminated or functionally silenced, thereby preventing the accumulation of pathogenic" are not logically supported by the references presented.

The authors should go through the references once more to ensure their claims are based on experimental data, the references are accurate and clearly indicate where the authors suggest their own model without direct experimental support.

Response to Reviewers: EMBOR-2025-62744V3

We sincerely thank the reviewers for their careful re-evaluation of our manuscript and for their highly constructive feedback during this second round of review. These insightful comments have allowed us to significantly refine the scientific precision and conceptual clarity of our manuscript. We believe that the corrections to the major comments have fully resolved all outstanding points. Please find below the point-by-point response to the questions by each referee:

Referee #1:

The authors have addressed most comments, but there are some areas that need revising for accuracy.

Comments on B cell anergy: lines 108. Self-reactive B cells activated through the BCR but failing to receive T cell help do not persist. Very much like foreign antigen activated B cells, they migrate to the T:B borders of secondary lymphoid tissues and if they fail to receive T cell help they die within 72 hours. This needs to be clarified. Tony Basten and Barbara Fazekas de St Groth demonstrated that if self-reactive antigen-activated (so called anergic) B cells receive T cell help, they survive and mount normal responses (PMID: 9710232; PMID: 8642340), indicating that functional inactivation is effectively lack of T cell help.

Response:
We thank the reviewer for this insightful comment. We agree that peripheral B cell anergy is functionally an operational state dictated by the absence of T cell co-stimulation rather than an immutable cell-death sentence.

As the reviewer rightly highlights, based on the seminal work by Basten and colleagues (Fulcher et al., 1996; Cook et al., 1998) using hen egg lysozyme transgenic models established these exact peripheral dynamics. Indeed, we have fully integrated this crucial clarification into our text (under subsection, *Central B cell tolerance*) to explicitly detail this outer PALS migration, the 72-hour survival window, and the operational nature of anergic B cell rescue, while formally citing both landmark papers.

Line 331 - Comments re "direct causality between sustained TLR7 signaling and autoimmunity remain to be established". This was established a few years ago with the demonstration that TLR7 GoF variants cause human lupus - Brown, G. J. et al. doi:10.1038/s41586-022-04642-z followed by multiple similar reports of TLR7 GoF variants or its chaperone UNC93B1 causing human systemic autoimmune disease:

- Menon, S. et al. Refractory Autoimmune Thrombocytopenia in an Infant with a De Novo TLR7 Gain-of-Function Variant. J Clin Immunol 45, 27, doi:10.1007/s10875-024-01824-4 (2024).
- David, C. et al. Gain-of-function human UNC93B1 variants cause systemic lupus erythematosus and chilblain lupus. J Exp Med 221, doi:10.1084/jem.20232066 (2024).
- David, C. et al. Interface Gain-of-Function Mutations in TLR7 Cause Systemic and Neuro-inflammatory Disease. J Clin Immunol 44, 60, doi:10.1007/s10875-024-01660-6 (2024).
- Mishra, H. et al. Disrupted degradative sorting of TLR7 is associated with human lupus. Sci Immunol 9, eadi9575, doi:10.1126/sciimmunol.adi9575 (2024).
- Al-Azab, M. et al. Genetic variants in UNC93B1 predispose to childhood-onset systemic lupus erythematosus. Nat Immunol 25, 969-980, doi:10.1038/s41590-024-01846-5 (2024).
- Han, X. et al. A novel *UNC93B1* gain-of-function mutation leads to TLR7 and TLR8 hyperactivation and systemic lupus erythematosus. medRxiv, 2024.2009.2011.24313360, doi:10.1101/2024.09.11.24313360 (2024).

- Stremenova Spegarova, J. et al. A de novo TLR7 gain-of-function mutation causing severe monogenic lupus in an infant. J Clin Invest 134, doi:10.1172/JCI179193 (2024).
- Tusseau, M. et al. Novel TLR7 gain-of-function variant and review of the associated disease spectrum. Journal of Human Immunity 2, doi:10.70962/jhi.20250199 (2026).
- Wolf, C. et al. UNC93B1 variants underlie TLR7-dependent autoimmunity. Sci Immunol 9, eadi9769, doi:10.1126/sciimmunol.adi9769 (2024).
- Zeng, Y. et al. Somatic gain-of-function mutation in TLR7 causes early-onset systemic lupus erythematosus. Ann Rheum Dis 84, 442-450, doi:10.1016/j.ard.2025.01.011 (2025).
- Parvaneh, N. et al. TLR7 F507S gain-of-function mutation presenting with early-onset SLE and hypertriglyceridemia: Response to JAK inhibition. Journal of Human Immunity 2, doi:10.70962/jhi.20250196 (2026).
- Sethumadhavan, A. et al. Identification of a novel TLR7 gain-of-function variant that underlies systemic lupus erythematosus. Journal of Human Immunity 2, doi:10.70962/jhi.20250194 (2026).

Response:

We thank the reviewer for pointing out this critical oversight. We completely agree that recent genetic discoveries have definitively established a direct causal relationship between enhanced TLR7 pathway activation and human systemic autoimmunity. Hence, we removed the statement questioning direct causality and have completely revised this section to highlight the definitive role of TLR7 gain-of-function (GoF) mutations and its chaperone UNC93B1 in driving human lupus and related autoimmune syndromes. Following the reviewer's comprehensive and valuable list of references, we have integrated these groundbreaking human genetic studies, including the seminal work on monogenic lupus (Brown et al, 2022) alongside subsequent reports on TLR7 and UNC93B1 hyperactivation syndromes, into our revised manuscript (subsection, "*Inflammatory milieu and checkpoint erosion*", second paragraph).

Line 428. Might be worth mentioning that TLR7 is required for the response to SARS-Cov2 (several papers showing male patients with LoF genetic variants in TLR7 develop more severe disease) - Asano et al., Sci. Immunol.6, eabl4348 (2021); Solanich et al., Front. Immunol.12, 719115 (2021); Butler-Laporte et al., PLOS Genet.18, e1010367 (2022).

***Response*:**

We thank the reviewer for this relevant suggestion. We completely agree that acknowledging the indispensable role of TLR7-dependent immunity in host defense against SARS-CoV-2 provides necessary and valuable context alongside its role in driving autoimmunity when hyperactivated.

We have fully accepted this suggestion and updated our manuscript text (now integrated in subsection *"**Systemic tolerance breakdown: SARS-CoV-2**")*. Following the reviewer's guidance, we have integrated the findings from all three recommended primary sources (Asano et al, 2021; Butler-Laporte et al, 2022; Solanich et al, 2021). The updated text highlights that rare, X-linked loss-of-function *TLR7* variants compromise crucial type I and II interferon (IFN) pathways, strongly predisposing young, otherwise healthy male patients to critical COVID-19 pneumonia.

Referee #2:

The revised review is much improved in content and style. Specifically, primary literature now supports their conclusions and statements.

Response:

We sincerely thank the referee for re-evaluating our manuscript and for the positive feedback regarding the revised text and style. We are very grateful for your valuable critiques during the previous round of review, which significantly sharpened our arguments and ensured thorough grounding in the primary literature.

Referee #3:
The authors present a review discusses mechanisms and pathways on how viral infection contribute to B-cell mediated autoimmunity. The manuscript reviews general central and peripheral B cell tolerance including EF and GC tolerance checkpoints. The authors aim to make a unifying point on how make viruses can subvert B cell tolerance. While the review is timely and unifies some of the findings in the field, there are a few reviews on published on the topic recently, with slightly different emphasis, such as Vinuesa, Grenov Kassiostis 2023 Science (DOI: 10.1126/science.adg6427), Cleaveer et al. Brain 2024 (DOI: 10.1093/brain/awad419).

Response:
We thank the reviewer for these excellent and highly relevant recent publications. We agree that positioning our manuscript relative to these works is essential to clarify our unique contribution to the field. We have revised our Introduction to explicitly integrate these references and delineate our conceptual framework from theirs. For example, Vinuesa et al. (Science 2023) mapped host-intrinsic, inborn genetic defects in nucleic acid-sensing pathways that drive chronic interferonopathies. In contrast, our review focuses on how wild-type viral infections environmentally engineer checkpoint failure in genetically typical hosts. Cleaver et al. (Brain, 2024) provides a comprehensive look at a single pathogen (HSV) within a specific compartment (i.e., CNS). In contrast, our manuscript shows a broad, comparative framework across systemic, tissue-restricted, and neurotropic infections to show how anatomical viral tropism dictates clinical landscapes of B cell-mediated autoimmunity. By adding these distinctions to the ***Introduction*** (concluding statements of third and fourth paragraphs), we have now clearly outlined its unique and complementary value to the existing literature.

While the manuscript has its merits, it suffers from several factual inaccuracies, poorly supported conclusions and inappropriate references to articles. A couple of examples:

Lines 159-160
Pillai & Cariappa 2009 do not suggest or demonstrate T-independent responses taking place in bone marrow. This is a blatant error and should be corrected.

Response:
We thank the reviewer for pointing out this citation error. We agree that the work by Pillai and Cariappa (2009, Nature Reviews Immunology, PMID: 19855403) we initially cited focuses primarily on the splenic commitment pathways of follicular versus marginal zone B cells. The demonstration of mature recirculating perisinusoidal B cells executing T-independent responses in the bone marrow was actually shown by the same group in an earlier seminal paper (Cariappa et al., Immunity, 2005, PMID: 16226505) and further discussed in Pillai and Cariappa (Immunol and Cell Biol, 2009, PMID: 19030017). We have amended the text to explicitly state “perisinusoidal B cells” instead of general FO B cells to ensure precise terminology. More importantly, we have updated the text with the correct citations.

Lines 187-188
"...the dark zone (DZ), where rapid proliferation, somatic hypermutation (SHM), and class-switch recombination occur, and..." and the Figure 2
The CSR switching would require T cell help. DZ is largely devoid of T cells. The idea that GC are the major site of CSR has been challenged by a finding that large proporsion of CSR occurs prior to GC (Roco JA, et al. Class-Switch Recombination Occurs Infrequently in Germinal Centers. Immunity. 2019 Aug 20;51(2):337-350.e7. doi: 10.1016/j.immuni.2019.07.001)

Response:
We thank the reviewer and completely agree with the reviewer's excellent point. The conventional view that class-switch recombination (CSR) is highly favored in the germinal center (GC) dark zone (DZ) has indeed been convincingly challenged. As established by Roco and colleagues (Immunity, 2019; PMID: 31375460), a vast majority of CSR events occur during initial T cell-B cell interactions prior to differentiation into mature GC B cells. This means that the DZ is primarily dictated for rapid clonal expansion and somatic hypermutation (SHM) rather than active isotype switching. Further, we also acknowledge that the DZ is anatomically distinct and is largely devoid of the T follicular helper cells required to provide the CD40L and cytokine signals that drive CSR.

Thus, we have revised our manuscript (first paragraph of subsection "***Canonical Germinal Center responses and immune checkpoints***") to remove the claim that CSR occurs in the dark zone and have explicitly integrated the pre-GC model of CSR with references to Roco et al. 2019. Furthermore, **Figure 2** has been modified to remove "CSR" from the DZ compartment, accurately reflecting the current spatial models of B cell differentiation and dynamics. We hope that these revisions have corrected the factual inaccuracy and align with the description of the DZ/LZ dynamics with modern literature.

Lines 209-220:
Inaccurate references throughout:
Mayer et al 2020 do not seem to demonstrate that germinal centers generate self reactive antibodies, but rather refer to earlier findings on this. In fact the message of the article seems to be rather that there is a checkpoint after GC reaction. Schwickert et al. 2011 do not investigate GC Tfh cells, but rather cues before establishing GCs. Merkenschlager et al 2021 do not seems to provide evidence for preferential selection for foreign antigen over autoantigens. Phan et al 2006 do not make distiction between autoantigens and foreign antigens. As a result, the claims of GC-intrinsic selecton against autoreactivity e.g. "GC reactions incorporate multiple, well-defined immune checkpoints that actively constrain self-reactivity" or "Together, these checkpoints ensure that SHM-induced reactivity is efficiently eliminated or functionally silenced, thereby preventing the accumulation of pathogenic" are not logically supported by the references presented.

Response:
We thank the reviewer for this rigorous critique. We completely agree that our original phrasing conflated general affinity selection mechanisms with intrinsic tolerance checkpoints against self-reactivity, and that several citations were misattributed. Specifically, we acknowledge that: (i) Mayer et al. (2020) demonstrated that autoreactive B cells can be tolerated within the GC proper. Additionally, they discovered a crucial post-GC checkpoint where autoreactive MBCs and plasma cells are removed by apoptosis. (ii) Schwickert et al. (2011) focus on T cell-mediated, affinity-dependent selection checkpoints regulating B cell entry into the GC at the T-B border, and not the dynamics of Tfh inside an established GC. (iii) Merkenschlager et al. (2021) investigated how the clonal behavior of Tfh repertoire remodels its own TCR specificities during a standard

response, rather than how Tfh discriminates foreign vs self-antigen presentation of B cells. (iv) Phan et al. (2006) and Victora and Nussenzweig (2022) describe general competitive affinity maturation and clonal competition on FDCs, without evaluating a self-versus foreign antigen distinction.

We have fundamentally restructured this section to decouple the concepts of general affinity maturation from peripheral tolerance. We now explicitly state that while GC selection operates primarily on antigen affinity, the elimination of somatically mutated, pathogenic autoreactive clones heavily relies on a stringent post-GC apoptotic checkpoint, aligning with the findings of Mayer et al., 2020. We have also corrected text referencing Schwickert et al. (2011) and removed the reference by Merkenschlager et al. which does not support the latest revision of this subsection.

The authors should go through the references once more to ensure their claims are based on experimental data, the references are accurate and clearly indicate where the authors suggest their own model without direct experimental support.

Response:
Besides the comments previously raised by the reviewer, we have also made a comprehensive audit of the full manuscript, including all the references, to ensure absolute precision of the statements and claims in all sections and subsections. We have made the following additional changes accordingly:

Introduction:

1. Ensure maximum scientific precision and clarification of the work by Wardemann and colleagues (PMID: 12920303) to specify that high-frequency autoreactivity is characteristic of the early pre-immune or immature pool, which is pruned down prior to establishing the mature naïve pool.
2. Refinement of the listing of tolerance checkpoints to clearly separate foundational processes (editing, deletion, and anergy) from the microenvironmental contexts in which they operate, namely GC and EF.

Central B cell tolerance

1. Exchanged the citation Victora and Nussenzweig (2022), which outlines mature germinal center dynamics rather than early bone marrow lymphopoiesis, with references that discussed foundational B cell development (Hardy and Hayakawa, 2001, PMID: 11244048; Cooper, 2015, PMID: 25656707).
2. Refinement of the phrasing around receptor editing to accurately reflect its role as a primary mechanism used to rescue autoreactive clones from negative selection pathways. The three principal routes were also reordered to reflect their occurrence in true biological sequence.
3. Clarified that cells with non-productive Ig gene rearrangements die by neglect at earlier precursor stages due to lack of tonic survival signaling, whereas clonal deletion explicitly deletes cells expressing productive, self-reactive BCRs.
4. Corrected our description of the microRNA-17~92/miR-19 axis based on Lai et al. (2016, PMID: 27481093) to accurately reflect that miR-19-mediated suppression of PTEN drives PI3K activation to promote positive selection while actively suppressing *Foxo1*-dependent receptor editing.
5. Reworded Figure 1 legend to match the precise cellular definitions in our revised manuscript. We have also updated Figure 1 to eliminate conceptual and visual ambiguities regarding central and peripheral checkpoints with the following changes: (i) removed

peripheral-to-central feedback loop to correctly show that peripheral selection leads exclusively to localized clonal deletion or anergy, and does not include receptor editing, which is strictly confined to the bone marrow microenvironment due to localized RAG1/2 reactivation constraints; (ii) updated box (1) nomenclature from "Negative selection" to "Central tolerance checkpoints".

6. Full harmonization of the "***B cell peripheral tolerance***" subsection for scientific accuracy and continuity with updated figures. Specifically, we corrected the transitional survival cytokine profile highlighting BAFF, updated our peripheral tracking statistics to precisely align with Wardemann et al. (2003), and replaced FDC-mediated clearance with the established mechanism of follicular exclusion.
7. Full harmonization of "***Canonical germinal center responses and immune checkpoints***" subsection with our updated Figure 2. Specifically, we corrected the following: (i) description of the intra-GC transcriptional wiring (Luo et al., 2018; PMID:29396161) to explicitly separate the dark-zone maintaining role of Foxo1 from the light-zone selection pathways of NF-κB and Myc and terminal differentiation toggle of IRF4; and (ii) corrected the anatomical terminology from "activated B cells re-entering the GC" to "germinal center B cells re-entering the dark zone".
8. Rephrased the paragraphs in subsection ***"Extrafollicular responses and immune checkpoints"*** to: (i) decouple the near-germline nature of healthy acute EF responses from the hypermutated nature of chronic immune EF expansion drive by Tph cells; (ii) expand the innate signaling axis to specify endosomal TLR7 and TLR9 as drivers of protective antiviral EF pathways; and (iii) demonstrate the differences between the GC and EF selection parameters and present a balanced view of EF responses in healthy or acute infections versus chronic autoimmune settings.
9. Revision of the subsection, "***Inflammatory milieu and checkpoint erosion***" to accurately capture the primary inhibitory axis utilized by Tfr cells, rectify the IL-2 dynamics, and integrate a recent literature demonstrating a direct B cell intrinsic IL-2 signaling
10. Enhanced the JEV conduit phrasing in subsection, "***antigen persistence and leakage***" to clarify that the lymphatic drainage functions as a peripheral priming conduit during neurotropic viral infection.
11. Polished ***Figure 3*** to ensure that the visual positioning of each panel is harmonized with the order of the revised manuscript.

Prof. Weiqiang Chen
Lee Kong Chian School of Medicine, Nanyang Technological University
Respiratory and Infectious Diseases
95 Nanyang Dr
Level 4
Singapore 636921
Singapore

Dear Prof. Chen,

Thanks for the submission of your further revised review manuscript. I have received the report from the referee that I asked to re-assess the article, which can be found at the end of this email. As you will see, the referee now supports publication of your review.

As I have now also received the final figures from the graphics designer, I am pleased to inform you that your review has been accepted for publication in EMBO reports.

Your manuscript will be processed for publication by EMBO Press. It will be copy edited and you will receive page proofs prior to publication. Please note that you will be contacted by Springer Nature Author Services to complete licensing information.

If you have any questions, please do not hesitate to contact the Editorial Office. Thank you for your contribution to EMBO Reports.

Yours sincerely,

Achim Breiling
Senior Editor
EMBO Reports

Referee #3:

The manuscript is greatly improved and satisfyingly addresses my key criticisms.

>>> Please note that it is EMBO Reports policy for the transcript of the editorial process (containing referee reports and your response letter) to be published as an online supplement to each paper. If you do NOT want this, you will need to inform the Editorial Office via email immediately. More information is available here: https://link.springer.com/partners/embo-press/editorial-policies#Peer%20review
